# Biofilms in Water Hoses of a Meat Processing Environment Harbor Complex Microbial Communities

**DOI:** 10.3389/fmicb.2022.832213

**Published:** 2022-02-14

**Authors:** Eva M. Voglauer, Benjamin Zwirzitz, Sarah Thalguter, Evelyne Selberherr, Martin Wagner, Kathrin Rychli

**Affiliations:** ^1^FFoQSI GmbH—Austrian Competence Centre for Feed and Food Quality, Safety and Innovation, Tulln, Austria; ^2^Unit of Food Microbiology, Institute for Food Safety, Food Technology and Veterinary Public Health, University of Veterinary Medicine Vienna, Vienna, Austria

**Keywords:** biofilm matrix, contamination source, food spoilage, facultative pathogenic bacteria, intracellular bacteria

## Abstract

Safe and hygienic water distribution is essential for maintaining product quality and safety. It is known that biofilms alter the appearance and microbial quality of water along the distribution chain. Yet, biofilms in water hoses throughout the food processing environment have not been investigated in detail. Here, microbial communities from water hoses and other environmental sites in contact with water, in addition to the source water itself, were studied in the meat processing environment. Biofilms were present in all water hoses as determined by the presence of bacterial DNA and biofilm matrix components (carbohydrates, extracellular DNA, and proteins). The microbial community of the biofilms was dominated by *Proteobacteria*, represented mainly by *Comamonadaceae* and *Pseudoxanthomonas*. Moreover, genera that are associated with an intracellular lifestyle (e.g., *Neochlamydia* and *Legionella*) were present. Overall, the microbial community of biofilms was less diverse than the water microbial community, while those from the different sample sites were distinct from each other. Indeed, only a few phyla were shared between the water hose biofilm and the source water or associated environmental samples. This study provides first insights towards understanding the microbiota of water hose biofilms in the food processing environment.

## Introduction

Ensuring water safety is an indispensable aspect of public health. In 2020, two billion people lacked safely managed water; therefore, the access to drinking water is included in the Sustainable Development Goals (SDG6). Even in countries with established water infrastructure, the water reaching the consumer is not guaranteed to be of high quality (United Nations, 2021).

There are various sources of contamination along the water transport chain, including physical (heavy metal particles), chemical (pesticides), and biological contaminants (microorganisms or their toxins; Bhagwat, 2019). Bacterial biofilms in water distribution systems, plumbing systems, and the last meters before water retrieval, e.g., water hoses, have recently been receiving particular attention (Carpentier and Cerf, 1993; Ren et al., 2015; Husband et al., 2016; Neu et al., 2019). They are responsible for the deterioration of drinking water quality, in terms of microbial safety and appearance, and the corrosion of pipes. Moreover, they decrease water carrying capacity and thereby lead to increased energy needs (Kumar and Anand, 1998; Wingender and Flemming, 2011; Kip and Van Veen, 2015; Husband et al., 2016). As water system biofilms develop in environments with low nutrient sources and high stresses, such as shear forces, disinfecting agents, and temperature variations, they are also of interest in studying bacterial adaption and resilience.

In various environments, biofilms have been detected in installations for water retrieval. In recent years, studies on biofilms in shower and garden hoses have highlighted the complexity and diversity at the last step in water distribution (Thomas et al., 2014; Soto-Giron et al., 2016; Proctor et al., 2016, 2018; Neu et al., 2019). The major cause for concern for biofilms in water hoses or water distribution systems is that bacteria or clusters thereof can detach, resulting in the contamination of water. Studies have shown that microorganisms from pipe biofilms appear in drinking water (among others Chan et al., 2019; Fish et al., 2020; Wingender and Flemming, 2004). Furthermore, the presence of pathogens in water hose biofilms has been reported, highlighting the potential health threat posed by these biofilms (Thomas et al., 2014; Soto-Giron et al., 2016).

In the food industry, the quality and safety of water are essential for maintaining product quality. Water is used for primary food production, during processing operations, as a food ingredient, and for cleaning and disinfection procedures (Kirby et al., 2003; Bhagwat, 2019).

In large-scale food production, water hoses are used to retrieve water. As in other water-associated environments, biofilms can form, potentially leading to contamination of food products or equipment. Within the food processing environment, two main usages for water hoses exist. Firstly, there are those used for cleaning and disinfection, where the water is typically hot and treated with disinfectants. Secondly, water can flow through hoses attached directly to certain machines. These hoses can be seen as food contact surfaces, as the water passing through them comes directly into contact with the food product itself or the surface on which the food product is processed. To date, these water hoses in the food processing environment have received little attention in regards to microbiological safety and quality investigations. In a recent study, we identified the nozzles of such water hoses to harbor multi-species biofilms (Wagner et al., 2020).

The overall goal of this study was to gain insight into biofilms inside water hoses used in a meat processing environment. Therefore, we characterized biofilms from seven water hoses in a meat processing facility that used unchlorinated water. The bacterial load and the presence of three matrix components (carbohydrates, proteins, and eDNA) were determined. We also examined the microbial community of the hoses *via* high-throughput 16S rRNA gene sequencing. Additionally, the microbial communities of the source water and various other environmental sites were characterized throughout the facility. Knowledge about the microbial communities of water hoses in the food producing environment can help to prevent and understand contamination events during production.

## Materials and Methods

### Sampling

**Figure 1:**
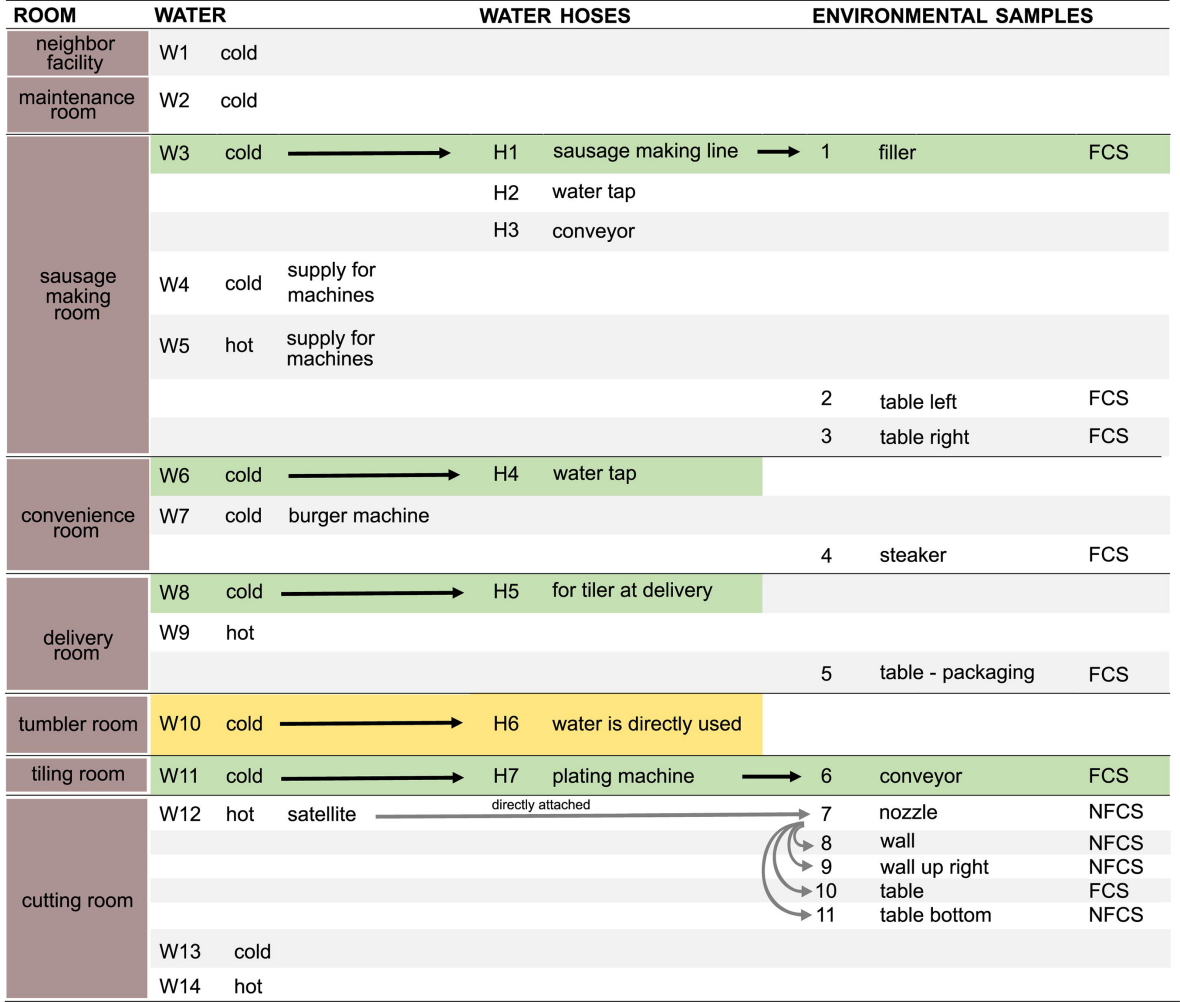
Schematic overview of samples. Water (*n* = 14, W1–W14), water hoses (*n* = 7, H1–H7), and environmental surfaces (*n* = 11, E1–E11) were sampled in different rooms. Arrows indicate a direct transfer of the water through the water hose to the environmental surface. Pairs of directly associated water, hoses, and environmental samples are highlighted in green. Rooms in which water is directly used for food production are highlighted in yellow. Food Contact Surface (FCS); Non-Food Contact Surface (NFCS). Detailed information on environmental samples are found in Supplementary Table 2.

Samples (Figure 1) were taken in July 2020 in an Austrian meat processing facility shortly after the end of production, after regular cleaning and disinfection. Three types of samples were taken: biofilms from water hoses (H, *n* = 7), water (W, *n* = 14), and environmental surfaces (E, *n* = 11).

#### Biofilm Sampling

Water hoses consisted of multiple layers, the innermost layer consisted of polyvinyl chloride (PVC). Water hoses were removed from the fittings and cut. One meter of each hose was put into a separate bag and transported to the laboratory. Biofilm samples were stored at 4°C until further processing within 24 h. Using a sterilized knife, 30 cm of the hose were cut. The hoses had an inner diameter of 1.2–1.5cm. The water hoses were sampled according to the scraper-flocked swab method (Maes et al., 2017; Supplementary Figure 1; Supplementary Table 1). A wetted swab was used to hydrate the biofilms before subsequent sampling with scrapers and swabs. The sampling devices were transferred into 10 ml .25x Ringer solution (B. Braun Austria GmbH). Next, 2 g of cation exchange resin (CER, described in detail in Wagner et al., 2020) was added to loosen the bacterial cells from the extracellular polymeric substances (EPS). Samples with CER were shaken for 20 min and centrifuged at 3320 rcf for 10 min. The supernatant (EPS-containing solution) was filtered using a .22 μm filter (Filtropur S0.2 Sarstedt AG& Co KG). The EPS samples and the cell pellets were frozen at −20°C until further processing.

#### Water Sampling

For each water sample, approximately 4 L of water were sampled in autoclaved wide-mouth bottles (Thermo Scientific™ Nalgene™ Wide-Mouth Lab Quality PPCO Bottles). Samples were taken before entering any connecting hose. The samples were transported to the lab at 4°C and the water was filtered through .22 μm filters (Millipore Type GS .22 μm, Merck Millipore; Supplementary Table 1). The filter papers were stored at −20°C until DNA extraction.

#### Sampling of Environmental Samples

Polyurethane sponges (SampleRight™ Sponge Sampler, World Bio Products) and flocked swabs (COPAN) were used for sampling of environmental sites (Supplementary Table 2). The type of sampling device was chosen upon accessibility of the surface. Hydrated polyurethane sponges were used to sample easy accessible areas. The respective area was horizontally and vertically wiped for several seconds, while using sterile gloves and a sterile size template. The swab was put back into the sterile bag and transported to the lab at 4°C. There, 10 ml of .25x Ringer solution was added and the sponge was squeezed extensively to gather the entire liquid. The liquid was then transferred to a 15 ml tube and centrifuged for 10 min at 3320 rcf. The supernatant was removed and the pellet was frozen at −20°C until DNA extraction.

For sites difficult to reach flocked swabs were used. Hereof, multiple devices [in addition to scrapers for larger surfaces areas (50–100 cm^2^)] were used to sample the respective area, by vertically and horizontally wiping for several seconds. The used swabs were added to 10 ml of .25x Ringer solution directly after sampling. The samples were stored at 4°C and processed within 24 h. In the laboratory, each sample was vortexed for 1 min and centrifuged for 10 min at 3220 rcf. The swabs and the supernatant were removed and the pellet was frozen at −20°C until DNA extraction.

Negative controls for all sampling types, solutions, and devices were included (Supplementary Table 3).

### DNA Extraction

DNA extraction was carried out using DNeasy PowerSoil Pro Kit (LOT: 166013851) as described previously (Wagner et al., 2020). Samples of each type were extracted at the same time. A negative control (DEPC-treated water) was included for each extraction round. The biofilm pellet was washed using PBS to remove the cation exchange resin (as described by Wagner et al., 2020). The filter papers (of the source water samples) were directly put into the PowerBead Pro tube. For determination of DNA concentration, the Spectrophotometer/Fluorometer DS-11 FX+ (DeNovix) was used.

### Matrix Evaluation

The presence of EPS components (carbohydrates, proteins, and eDNA) of the water hose biofilm samples was determined. A detailed description on these methods was recently published (Wagner et al., 2020). In short, an aliquot of 1 ml was used for carbohydrate determination using a phenol-sulfuric acid plate assay. The limit of quantification was 79 μg/10 ml glucose equivalents. Aliquots of 1 ml were used for an overnight protein precipitation using TCA/acetone. The precipitated samples were loaded into an SDS-PAGE (15%) and subsequently stained using silver-staining. Aliquots of 500 μl were precipitated using Na-acetate and ethanol, and quantified using the Spectrophotometer/Fluorometer DS-11 FX+ (DeNovix). For each precipitation and assay controls (i.e., glucose for carbohydrates, BSA for proteins, and salmon sperm DNA for eDNA) were included. Mean values and standard deviations were calculated in R and visualized using ggplot2 in R (Wickham, 2016).

### Enumeration of Bacterial Load Using qPCR

The total bacterial load, expressed as bacterial cell equivalents (BCE), was determined as previously described (Dixon et al., 2019). A quantitative real-time PCR (qPCR) using the primers 357-F(5′-CCT ACG GGA GGC AGC AG-3′) and 518-R(5′-ATT ACC GCG GCT GCT GG-3′) to target the 16S rRNA gene (Muyzer et al., 1993) was used (standard curve 1.77 to 6.77 log BCE, primer efficiency 102.8–107.08%). The reaction mixture consisted of 1x Brilliant III Ultra-Fast SYBR® Green qPCR Master Mix with Low ROX (Agilent), 250 nM of forward and reverse primer, and 5 μl of sample in a 25 μl reaction. The samples were analyzed in duplicate using the Stratagene Mx3000P qPCR System with an initial denaturation step at 95°C for 3 min. Amplification was carried out in 40 cycles of 3 s at 95°C and 20 s at 60°C. A melting curve was performed to identify amplicon specificity (95°C for 1 min, 60°C for 30 s, and 95°C for 30 s). In each run, a negative control (DEPC-treated water) was included (Supplementary Table 3). The evaluated copy number of the qPCR control and the respective kit extraction control were subtracted from the respective samples before extrapolation of the total BCE. An average of five 16S rRNA gene copies per cell was considered, as estimated using the database for ribosomal RNA operon variation in bacteria and archaea, rrnDB (Větrovský and Baldrian, 2013; Stoddard et al., 2015). Results were visualized using ggplot2 in R (Wickham, 2016).

### 16S rRNA Gene Sequencing, Processing, and Analysis

Amplicon library generation, quality control, and sequencing were performed by the Next-Generation Sequencing Facility at Vienna BioCenter Core Facilities (VBCF), member of the Vienna BioCenter, Austria. The V3V4 region of the 16S rRNA gene was amplified using the primers S-D-Bact-0341-b-S-17 F(5′-ctctttccctacacgacgctcttccgatct CCTACGGGNGGCWGCAG-3′) and S-D-Bact-0785-a-A-21 R(5’-ctggagttcagacgtgtgctcttccgatctGACTACHVGGGTATCTAATCC-3′; Klindworth et al., 2013). Sequencing was done on an Illumina MiSeq sequencing platform with a 300 bp paired-end read protocol.

Primer and illumina adapter sequences were trimmed from the raw sequence reads with trimmomatic v0.36 (Bolger et al., 2014). The rest of the sequence processing and quality control was performed in R v3.6.2 using the DADA2 pipeline v1.14 (Callahan et al., 2016; R Core Team, 2020). Briefly, reads with a maximum number of expected errors greater than 2 were removed and the remaining reads were truncated where the average phred quality score dropped below 30 (positions; fwd: 220 and rev: 200). The DADA2 sample-inference algorithm and the following merging of the forward and reverse reads were run with default parameters. Then, chimeras were identified and removed with the “removeBimeraDenovo” command using the consensus method. The remaining reads were annotated to the SILVA SSU database release 138 with a minimum bootstrap threshold of 50 (Quast et al., 2013). Annotated amplicon sequence variants (ASVs) with less than five counts in total were removed from the dataset before continuing analysis. Finally, potential contaminant sequences were identified with the R package “decontam” using the prevalence method, which compares the prevalence of each sequence in samples to the prevalence in negative controls (Davis et al., 2018). Here, the probability threshold in the “isContaminant” command was set to .5. A total of 309 ASVs were designated as potential contaminants and removed from the dataset (Supplementary Table 4).

Initial data exploration, and basic microbial community analysis, was conducted using the R package “phyloseq” and a dataset rarefied to the minimum sample size (5,474 sequences; McMurdie and Holmes, 2013). Alpha diversity indices were calculated and compared with vegan v2.5–6 (Oksanen et al., 2019) with pairwise comparisons using Wilcoxon rank sum tests and Benjamini-Hochberg adjustment for values of *p*. The dissimilarity in community composition was visualized in ampvis2 v2.6.6 by means of a principal coordinates analysis based on Bray-Curtis dissimilarities as a distance measure (Andersen et al., 2018). Due to not normal distribution of these data and residues, the beta diversity was further assessed by applying a permutational multivariate analysis of variance (PERMANOVA, formerly nonparametric MANOVA) with the adonis function and 5,000 permutations. The relative abundances of individual taxa were calculated and illustrated as barplots (Phyla) or heat maps (Top 50 ASVs) in “phyloseq” and “ampvis2,” respectively.

## Results

### All Water Hoses Harbored Biofilms

**Figure 2:**
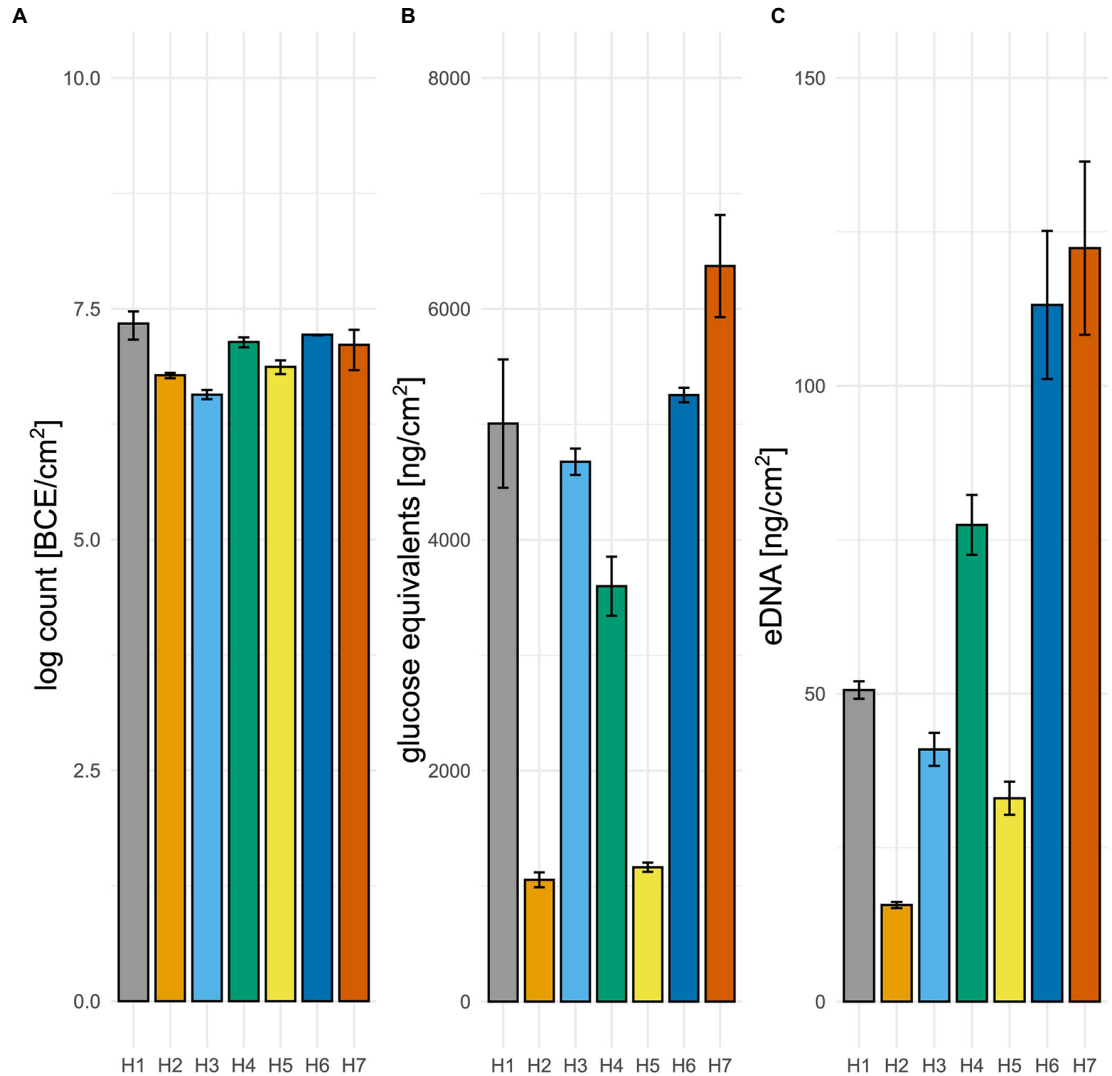
Water hose biofilms. **(A)** Number of bacterial cell equivalents in biofilms was determined using a qPCR assay targeting the 16S rRNA gene. **(B)** Matrix polysaccharides were determined using a phenol-sulfuric acid method. **(C)** The amount of eDNA was spectroscopically measured. Mean values and standard deviation are given of at least two individual measurements.

All seven water hoses (H1–H7) used in a meat production environment for machines or direct water retrieval had visible biofilms on their inside (Supplementary Figure 1). The bacterial load of all water hose biofilms was above 6.6 log BCE/cm^2^ (Figure 2A, minimum H3 6.6 ± .1 log BCE/cm^2^), as determined by quantitative PCR. The highest bacterial cell equivalent count has been detected in the biofilm of water hose H1 (7.3 ± .1 log BCE/cm^2^). The chemical characterization of the biofilm matrix confirmed the presence of carbohydrates, eDNA, and proteins in all water hoses (Figures 2B,C, proteins: Supplementary Figure 2). The mean matrix carbohydrate load was 3,874 ng glucose equivalents/cm^2^ (range from 1,054 ± 65 ng/cm^2^ in H2 to 6,371 ng/cm^2^ ± 195 ng/cm^2^ in H7). A mean eDNA load of 64.7 ng/cm^2^ (range from 15.7 ± .5 ng/cm^2^ in H2 to 122.4 ± 13.2 ng/cm^2^ in H7) was detected. Two of the hoses (H2 and H5) had a notable lower amount of carbohydrates than the other water hoses (Figure 2B, 1,055 ± 65 and 1,163 ± 40 ng/cm^2^, respectively). Additionally, a lower eDNA amount was observed in H2 (Figure 2C, 15.7 ± .5 ng/cm^2^). The highest biofilm matrix loads were observed in H7 with levels of 6,371 ± 443 ng/cm^2^ glucose equivalents and 122.4 ± 13.2 ng/cm^2^ eDNA.

**Figure 3:**
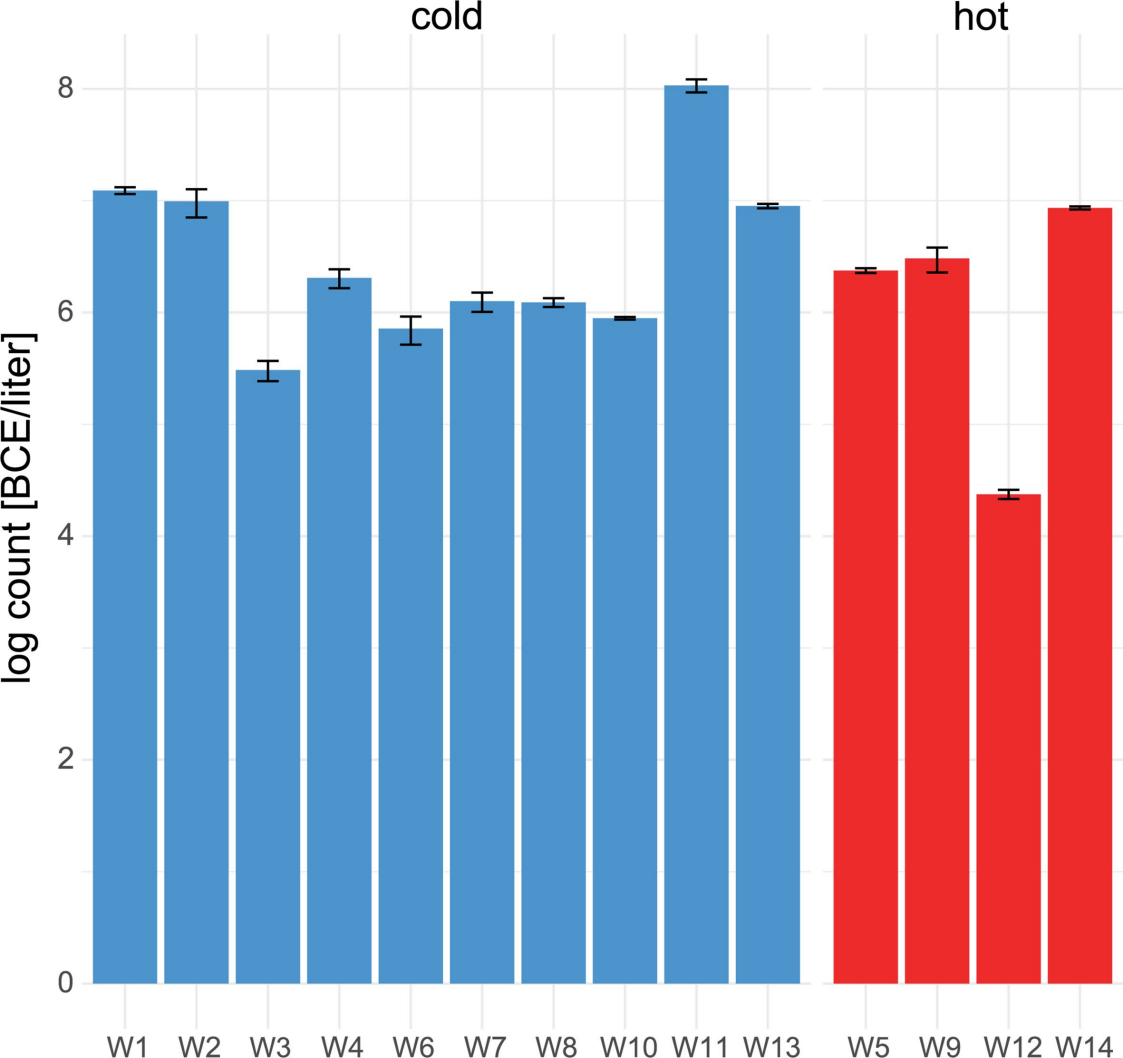
Number of bacterial cell equivalents in water. qPCR assay targeting the 16S rRNA gene was used to determine bacterial cell equivalents in cold (blue) and hot (red) water. Mean values and standard deviation of duplicate measurements are shown.

Within water samples, the mean bacterial cell equivalents were slightly higher in cold water (*n* = 11, 6.4 ± .7 log BCE/l) than in hot water (*n* = 4, 6 ± 1.1 log BCE/l, Figure 3A). The lowest bacterial load was seen in W12 (4.4 ± .04 log BCE/l), which is part of the cleaning system using hot water. Overall, a low bacterial cell load was detected at the environment sites (Supplementary Table 2). Here, the food contact surfaces (*n* = 6) had a mean bacterial cell equivalent load of 1.64 ± .59 log BCE/cm^2^ and non-food contact surfaces (*n* = 5) of .5 ± 1.2 log BCE/cm^2^.

### Microbial Communities in Water Hose Biofilms

In total, 4,581,962 16S rRNA gene sequences passed quality control, and 9,336 ASVs were classified, resulting in 101,821 ± 76,557 sequences per sample on average.

**Figure 4:**
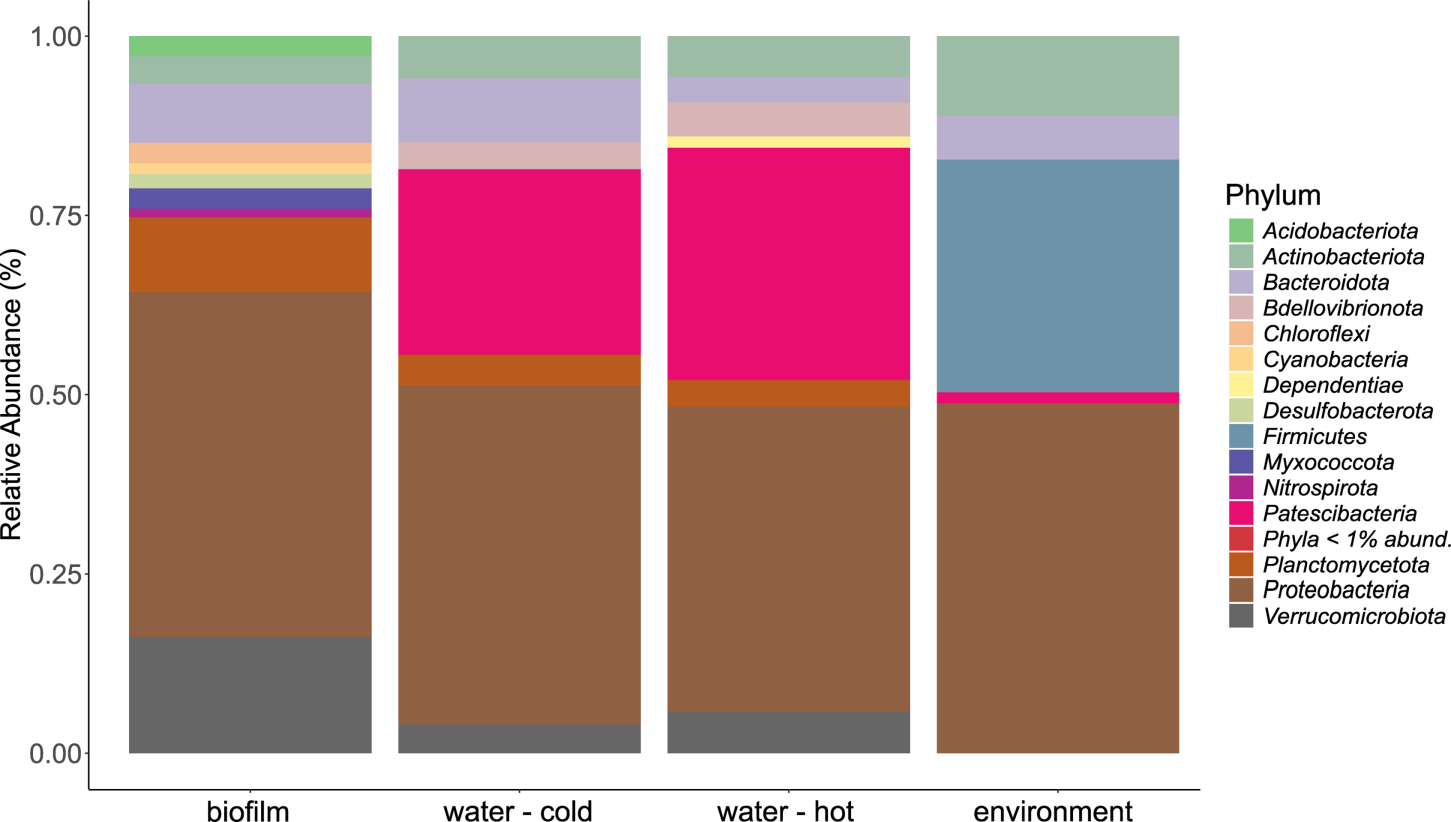
Mean phyla distribution obtained by 16 s rRNA gene analysis from microbial communities of water hose biofilms, water, and environmental samples.

**Figure 5:**
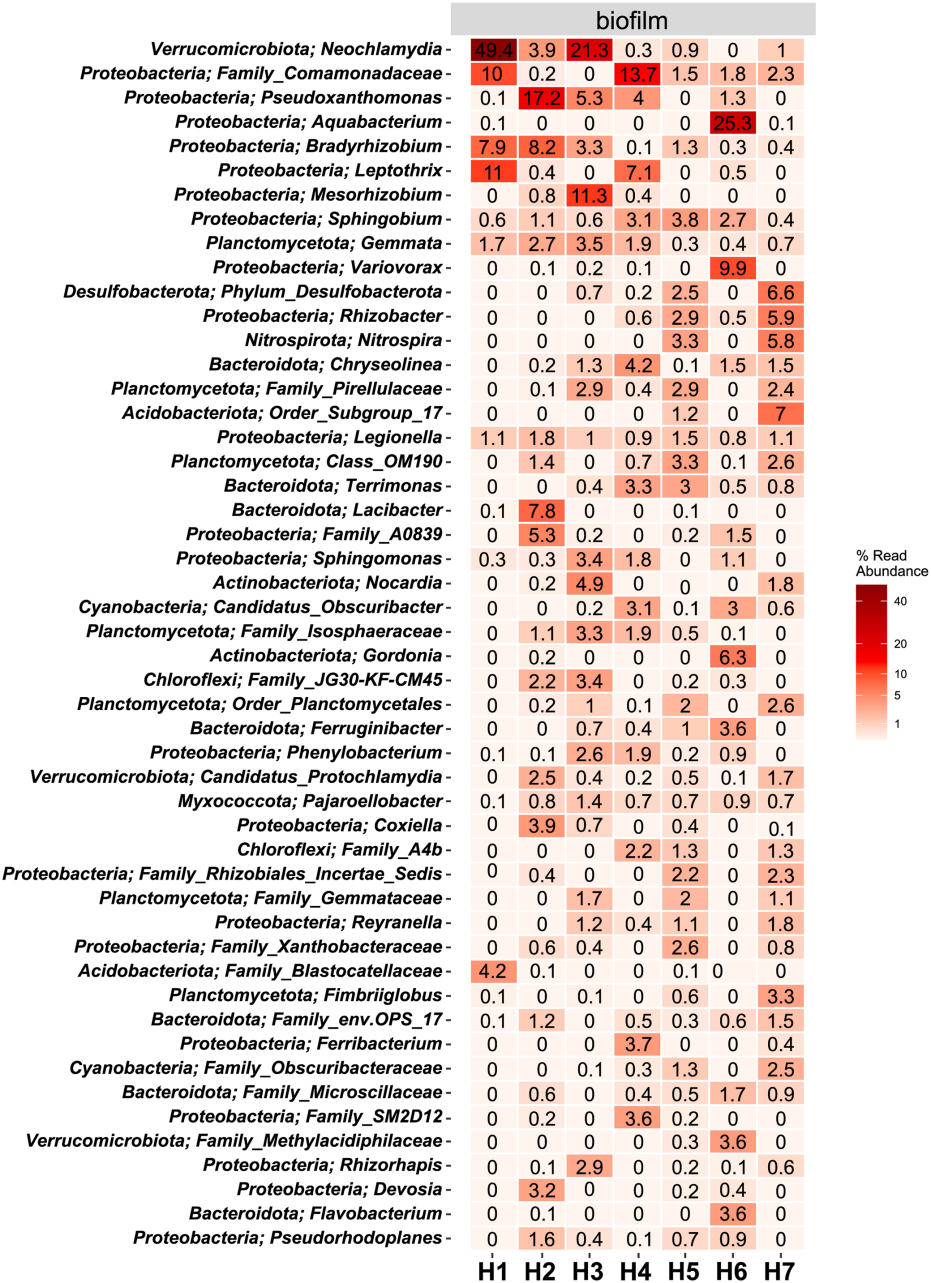
Heat map of the top 50 most abundant genera (and their belonging phylum) in biofilm samples. The average ASV counts from replicate 16S rRNA gene libraries for each category are shown. Numbers represent the read abundance in %.

The microbial community in water hose biofilms (Figures 4, 5; Supplementary Figures 3 and 4) was dominated by *Proteobacteria* ranging from 30.2% (H7) to 62.8% (H5). Within this phylum, the most abundant genera were related to the *Family_Comamonadaceae* (present in five hoses at abundances >1%) and *Pseudoxanthomonas* (in H2, H3, H4, and H6 > 1%). The phylum *Verrucomicrobiota* accounted for 14.3% mean relative abundance in water hose biofilms (ranging from 3.7% in H4 to 49.4% in H1) with highest levels of *Neochlamydia*, which were present in four hoses ≥1%, predominantly in H1 (49.4%) and H3 (21.1%). The third most abundant phylum in water hose biofilms was *Planctomycetota* [mean relative abundance of 9.5%, ranging from 1.8% (H1) to 16.2% (H5)] represented by sequences associated with the genus *Gemmata* (present in four hoses >1.7% abundance), and the family *Pirellulaceae* (present in 3 hoses ≥2.4%).

In water hose H6, the genus *Aquabacterium* was highly abundant (25.3%), which was not the case in the other hoses. *Legionella*, belonging to the phylum of *Proteobacteria*, could be detected in all biofilm samples at abundances ≥.8%.

### Microbial Communities in Water Samples

Also in source water samples, *Proteobacteria* (45.7% in cold and 41% read abundance in hot water, respectively) presented the most abundant phylum. Within *Proteobacteria*, the *Family_SM2D12* was present in all water samples at relative abundances >.7%. The next frequent phylum was *Patescibacteria* (23.3% in cold and 29.5% read abundance in hot water, respectively; Figure 4). Within *Patescibacteria*, the *Order_Saccharimonadales* (>51% in cold water, >.5% in all hot water samples) and the *Order_Candidatus_Uhrbacteria* (present in all water samples >.3%) were the most abundant genera (Supplementary Figure 3).

Sample W1, taken from the neighbor company having the same water supply, and W2, taken from the main service room and representing the first entry site of the water in the meat processing facility, differed from each other. Especially, the genera *Order_Saccharimonadales* (10.2% vs. 1.9% relative abundance), *Family_LWQ* (7.9% vs. .5% relative abundance), *Nevskia* (8.5% vs. 0% relative abundance), *Novosphingobium* (.1% vs. 17% relative abundance), and *Neochlamydia* (.9% vs. 5% relative abundance) showed different abundances between these two samples. In water W11, the uncultured phylum *KCM-B-112* was predominant (32.5% relative abundance); however, this phylum was not observed in high relative abundances in other water samples.

Cold and hot water differed significantly (*p* < .05) in the abundance of certain genera (Supplementary Figure 6). The presence of *Sphingomonas* was more prominent in hot water compared to cold water. In cold water several genera, such as *Rhodobacter*, *Legionella*, *Kocuria*, *Pseudomonas*, *Brochothrix* etc., were more abundant. Overall, the hot water samples were less diverse than the cold water samples (6,111 ASVs in hot vs. 13,913 ASVs in cold water, respectively).

### Microbial Communities of Environmental Samples

Within environmental samples, less phyla with >1% read abundance were present (n = 9) than in biofilm samples (Figure 4, *n* = 11). The most prominent phylum was again *Proteobacteria* (53.7%), followed by *Firmicutes* (23.8%) and *Actinobacteriota* (12.2%; Figure 4). The most abundant genera were *Pseudomonas* (11.7% mean relative abundance), *Photobacterium* (6.2% mean relative abundance), *Roseomonas* (5% mean relative abundance), and *Acinetobacter* (4.4% mean relative abundance). Single sites were dominated (relative read abundance >20%) by single genera, such as the filler (E1, FCS) by *Roseomonas*, the steaker (E4, FCS) and the bottom of the table (E11, NFCS) by *Pseudomonas,* and the wall of the cutting room (E8, NFCS) by *Photobacterium* (Supplementary Figure 3).

### Differences in Community Structure Between Biofilms, Water, and Environmental Samples

**Figure 6:**
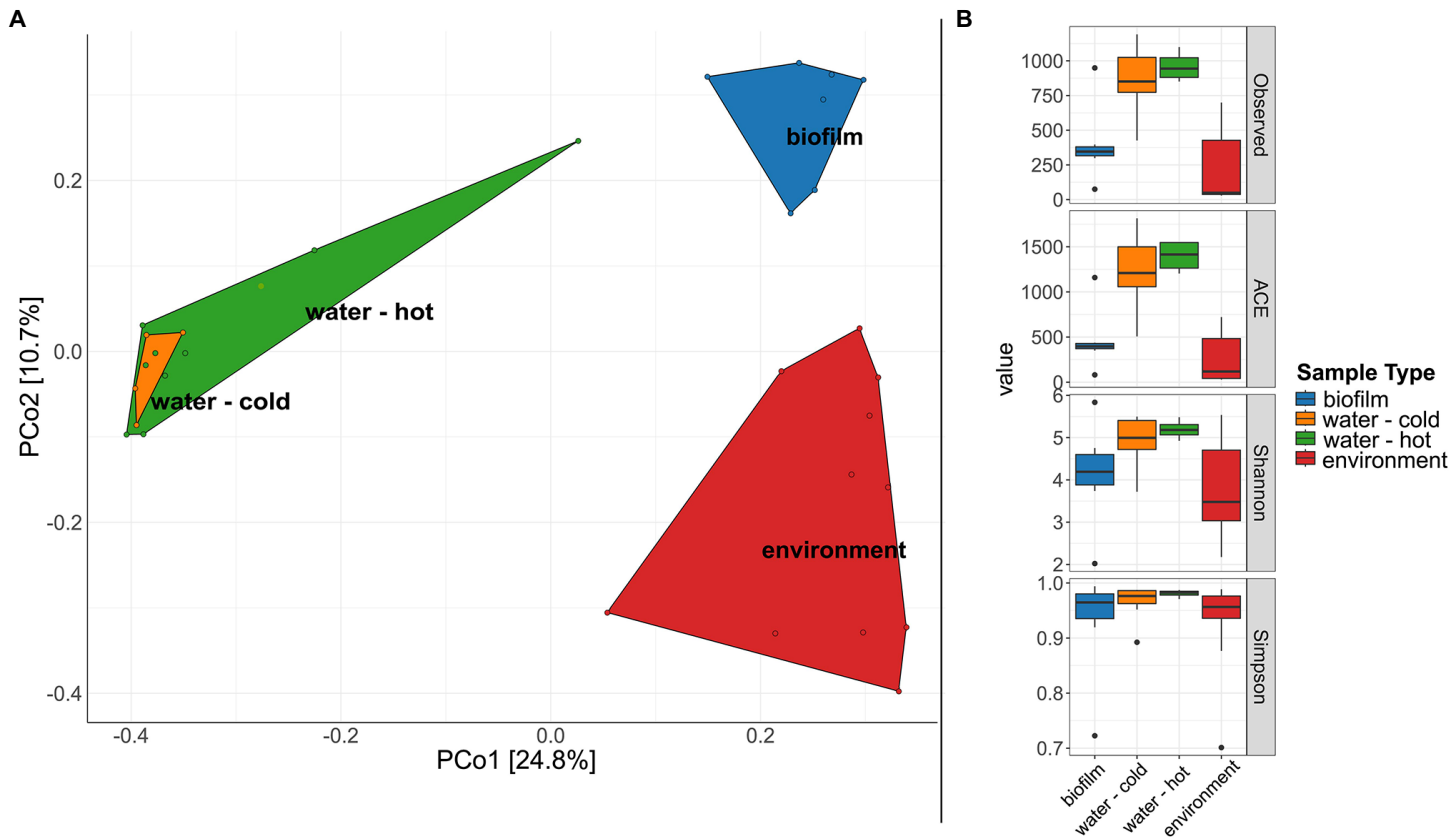
Diversity of microbial communities in water, water hoses, and in the environment. **(A)** Principal coordinates analysis (PCoA) plot showing clustering of different sample types. **(B)** Alpha diversity indices (Observed, ACE, Shannon, Simpson) for bacterial communities of the different sample types: biofilms from water hoses (blue), water [cold (orange) and hot (green)], and environmental samples (red).

A significant difference in microbial communities was revealed by beta-diversity analysis (Figure 6A, PERMANOVA; beta-diversity metric, *R*^2^ = .107, *df* = 1, *p* = .0002). The respective sample types clustered, whereas hot and cold water could not be distinguished. The microbial biodiversity was estimated within samples using different alpha diversity indices (observed species, ACE, Shannon, and Simpson index, Figure 6B; Supplementary Table 5). Water samples were overall the most diverse with 862.5 and 959.5 observed ASVs for cold and hot water, respectively. Observed species were significantly lower in biofilms compared to water; however, no significant differences were observed for Shannon and Simpson indices (*p* > .05%). In total, the three different sample types, hose biofilms, water, and environmental sites shared 17 ASVs (ASVs being present in at least seven samples of biofilms and 10 for the other groups with a minimum relative abundance of .01 in each sample type), resulting in a combined relative abundance of 3.3% (Supplementary Figure 5). Taxonomic classification revealed the genera *Brevundimonas*, *Pseudomonas*, *Legionella*, and *Sphingomonas* among represented genera (Supplementary Table 6).

The hoses shared several genera with the respective water, yet no clear trend between the associated water and the hoses can be observed (Figure 5; Supplementary Figure 3). In the sausage making room, the water hose H1 and the linked source water W3 shared the first and the second most abundant genera *Leptothrix* and *Family*_*Comamonadaceae* [H1 (11% and 2.4%, respectively); W3 (10% and 1.2%, respectively)]. Yet, the dominating *Neochlamydia* (49.4% read abundance) was only observed in the water hose and not in the source water. A limited number of genera were shared between the environmental sites and their linked hoses and water (Supplementary Figure 4). In the sausage making room, *Sphingobium* was the only phylum that was detected in H1, W3, and E1 (.6%, 1.4%, and .8% read abundance, respectively). *Family*_*Comamonadaceae* and *Sphingobium* reads were present in most hoses and waters, yet they were hardly detected at environmental sites. Water hose H1 and its associated environment E1 shared *Sphingomonas* sequences, in addition to *Sphingobium*, which was also present in the water,

In the tiling room, H7 shared the phylum *Family*_*Microscillaceae* with its source water W11 (10.9% in W11; .9% in H7), yet this phylum could not be detected at the associated environmental site E6. The water hose H7 and its connected environmental site E6 (the conveyor) shared reads of *Family_ Comamonadaceae* (2.3% vs. .7%), *Legionella* (1.1% vs. 2.5%), and *Mycobacterium* (.3% vs. 2.5%) at abundances greater than .1% in the hose and associated environmental site.

In the tumbler room, the water (W11) and the associated hose (H6) shared three phyla (abundance ≥1%). Two phyla were shared between the water and the hose (abundance ≥1%) in the convenience room (W6-H4) and the delivery room (W8-H5).

## Discussion

The presence of biofilms in water-associated equipment leads to several consequences, such as the deterioration of drinking water quality, the possible transmission of bacteria, corrosion of pipes, and a decrease of water carrying capacity. Studies investigating water hose biofilms are rare and limited to specific environments. Biofilm formation in water hoses has been documented in the hospital setting (Soto-Giron et al., 2016), garden hoses (Thomas et al., 2014), and domestic shower hoses (Moat et al., 2016; Proctor et al., 2016, 2018; Neu et al., 2019). Safe water is indispensable for public health and water quality has a huge impact on food quality and security (Bhagwat, 2019). Water is known to be colonized by bacteria from biofilms on contacting materials, such as pipes (Chan et al., 2019) and drinking water distribution systems (Wingender and Flemming, 2004). This is a crucial concern in food production, as water is used in various processing steps. Within the food processing environment, this is the first study that focuses on the characterization of biofilms in water hoses. We demonstrated that different water hoses, used in the meat processing context for many years, harbored biofilms with a complex and unique microbial community. Biofilms visible to the naked eye could be observed in all water hoses, comparable to other studies dealing with materials in contact with water (Thomas et al., 2014; Neu et al., 2018, 2019; Proctor et al., 2018). The presence of matrix components (carbohydrate, proteins, and extracellular DNA) in the biofilm could be confirmed. These analyses were included to confirm the presence of biofilms, as they are defined to be composed of both microorganisms and matrix. Moreover, these analyses showed that the biofilm matrix of water hose biofilms harbor different amounts of carbohydrates, proteins, and extracellular DNA, which speaks to the complexity of these biofilms. The bacterial load of these water hose biofilms was comparable to those identified in studies focusing on biofilms in shower hoses and drinking water distribution network biofilms (Wingender and Flemming, 2004; Proctor et al., 2018). *Proteobacteria* were predominantly present in all water hose biofilms, its source water and associated environments, which is in line with previous studies focusing on biofilms from water distribution and plumbing systems, as well as water contacting materials (Hong et al., 2010; Pinto et al., 2012; Ren et al., 2015; Moat et al., 2016; Proctor et al., 2016, 2018; Neu et al., 2018; de Sotto et al., 2020).

The microbial community of the water hose biofilms in the meat processing environment was diverse and differed between hoses collected in the same room. In all rooms, opportunistic pathogen containing genera, such as *Neochlamydia*, *Legionella*, or *Pseudomonas*, have been detected in different abundances. *Neochlamydia*, an obligate intracellular symbiont of amoebae, was highly present in the sausage making room, especially in water hose H1 and water hose H3. In the other rooms, this genus showed low abundance. In the tiling room, *Neochlamydia* was present in both the water (W11) and the linked water hose (H7), which could indicate a possible transmission. Another facultative intracellular pathogenic genus detected in all water hoses and the source water was *Legionella*, whose presence has been described in domestic shower hoses (Proctor et al., 2016, 2018), garden hoses (Thomas et al., 2014), and cooling towers (Tsao et al., 2019). The presence of *Legionella* has previously been reported to correlate with total cell counts (Proctor et al., 2018). Therefore, thick biofilms might act as a means of proliferation for *Legionella* (van der Kooij et al., 2017). The presence of facultative and obligate intracellular bacteria in these water hoses indicate the presence of protozoa in the water hose biofilms, as reported in a previous study on garden water hoses (Thomas et al., 2014). The role of amoebae in water-associated biofilms has already been investigated (Thomas et al., 2010, 2014; Morsy et al., 2016; Proctor et al., 2018; Taravaud et al., 2018; Shaheen et al., 2019), yet their definite role in the transfer of pathogenic bacteria in water-associated environments remains to be elucidated.

The detection of several other genera is in line with publications investigating different water-associated biofilms. *Pseudoxanthomonas* was also detected in hospital shower hoses (Soto-Giron et al., 2016), *Sphingobium* in drinking water distribution system (Fish et al., 2020) and hospital shower hoses (Soto-Giron et al., 2016), and *Bradyrhizobium* in hospital shower hoses (Soto-Giron et al., 2016), in domestic shower hoses (Moat et al., 2016; Proctor et al., 2018), in laboratory studies (Neu et al., 2019; Fish et al., 2020), and bath toys (Neu et al., 2018). *Sphingomonas*, which was present in three water hoses of the meat processing environment at relative abundances >1%, was also described to be present in hospital shower hoses (Soto-Giron et al., 2016), in domestic shower hoses (Moat et al., 2016; Proctor et al., 2018), and laboratory studies (Neu et al., 2019), as well as biofilms on plastic bath toys (Neu et al., 2018).

In a previous study, focusing on the detection of biofilms in the meat processing environment, we cultivated meat-spoilage bacteria, such as *Microbacterium*, *Stenotrophomonas*, *Brochothrix*, and *Psychrobacter*, from biofilms on water hose nozzles (Wagner et al., 2020). In the water hose biofilms of the present study, we also detected *Pseudomonas*, *Microbacterium*, and *Psychrobacter*. *Pseudomonas* and *Microbacterium* in two water hoses, and *Pseudomonas* in the supplying water (W3 and W11, respectively). However, other genera linked to meat spoilage, such as *Lactobacillus*, *Acetobacter*, *Kocuria*, *Lactococcus*, *Stenotrophomonas*, and *Carnobacterium* (Maes et al., 2019), have not been detected in water hose biofilms. Within a laboratory-based study, we could demonstrate that isolates from the genera *Microbacterium* and *Stenotrophomonas* were able to form mono-species biofilms on stainless-steel slides under conditions mimicking the food processing environment (Wagner et al., 2021). Additionally, we analyzed the microbial composition of water, where five samples were directly associated with the water hoses. The 16S rRNA gene load from the water was comparable with those identified by Waak et al. in non-treated water (Waak et al., 2019). The microbial community of the water had a higher species richness than water hoses, which is consistent with other studies (Proctor et al., 2018). Prominent taxa within the water included the *Order_Saccharimonadales*, the *Order_Candidatus_Uhrbacteria*, and *KCM-B-112*. The fact that representatives of these groups have not been cultivated so far highlights the need of further efforts to cultivate organisms from water distribution systems. The hoses, which had been installed for several years, and their associated water shared only a few phyla (e.g., *Leptothrix*, *Family_Comamonadaceae* and *Sphingobium*). The dominant taxa of the water and the respective water hose biofilm were different. This was also observed in other studies (Waak et al., 2019).

We further observed that the hot water samples were less diverse than the cold water samples, which is in line with a recent study (Proctor et al., 2018). Water poses a risk for food contamination when harboring spoilage bacteria. Previously, the presence of coliforms in wash water for milking equipment was reported to be a risk factor for contamination (Perkins et al., 2009). In this study, ASVs linked to meat-spoilage organisms, such as *Pseudomonas* (ASV101, ASV912), *Brochothrix* (ASV37), and *Kocuria* (ASV68), have been more frequently found in cold than in hot water, indicating the importance of water temperature. Furthermore, *Legionella* and *Mycobacterium* have been identified in the water. Even though these bacteria might not pose a problem in terms of food contamination, they might pose a safety issue for workers that inhale the aerosols produced during cleaning and disinfection (Wingender and Flemming, 2004, 2011). These are important findings for the management of water safety in the food industry for both the workers and consumers health.

Despite the water showing high levels of bacterial cell equivalents, the water itself is not the only source for microorganisms in water hose biofilms. Aerosols are capable of transferring microorganisms to water hoses. A study using a GFP-tagged *Pseudomonas putida* strain showed that this motile bacterium was able to colonize the inside, and the outside of a water hose, after being transferred to the hose by aerosols resulting from high pressure water cleaning (Gagnière et al., 2006). This observation together with the evaluation of water hoses as potential contamination sources supports the notion that intervention strategies to prevent colonization of water hoses have to be taken. For example, hindering of contamination by aerosols could be done by storing the exposed openings in disinfecting solutions (Gagnière et al., 2006). The colonization of hoses by microorganisms derived from the water source could be facilitated by different approaches, such as (i) filtering of the water before it enters the hose, (ii) using materials that hinder biofilm formation in hoses, and (iii) the frequent replacement of water hoses.

Additionally, we investigated environmental samples, which showed a low microbial load. Few phyla were shared between the hoses and the associated environmental sites. *Pseudomonas*, *Photobacterium*, *Acinetobacter*, and *Roseomonas* were more abundant at the environmental sites compared to the biofilm or water samples. With the exception of the *Roseomonas*, these have already been described as being most abundant in the environment of another meat processing facility (Zwirzitz et al., 2021), indicating that these microbes might have adapted to cleaning and disinfection strategies, and other characteristics of the food processing environment (e.g., cool temperatures). Nevertheless, the observation of 16S rRNA gene sequences of these genera does not allow us to say anything about whether these microbes, are viable as no cultivation-based approaches were applied in this study.

## Conclusion

This study demonstrates the complexity of biofilms in water hoses in a meat processing environment. We showed that different water hoses linked to meat processing equipment harbored biofilms with unique microbial communities and different matrix amounts. This study only represents a first snapshot on this issue. Further studies are needed to understand the role of water and water hoses as contamination source in the food producing environment and the role of certain environmental characteristics, such as nutrient sources, materials (e.g., Proctor et al., 2016), temperature, and time, on the formation of biofilms in water hoses.

## Data Availability Statement

The datasets presented in this study can be found in online repositories. The names of the repository/repositories and accession number(s) can be found at: https://www.ebi.ac.uk/ena, PRJEB49015.

## Author Contributions

EV, KR, ES, and MW conceived and designed the study. Sampling was performed by EV and KR. EV and ST performed the sample processing. BZ developed bioinformatics pipelines. BZ, EV, and ST performed data analysis. BZ, EV, and KR wrote the manuscript. All authors contributed to the article and approved the submitted version.

## Funding

This work was created within a research project of the Austrian Competence Centre for Feed and Food Quality, Safety and Innovation (FFoQSI). The COMET-K1 competence center FFoQSI is funded by the Austrian federal ministries BMK, BMDW, and the Austrian provinces Lower Austria, Upper Austria, and Vienna within the scope of COMET—Competence Centers for Excellent Technologies. The program COMET is handled by the Austrian Research Promotion Agency FFG.

## Conflict of Interest

The authors declare that the research was conducted in the absence of any commercial or financial relationships that could be construed as a potential conflict of interest.

## Publisher’s Note

All claims expressed in this article are solely those of the authors and do not necessarily represent those of their affiliated organizations, or those of the publisher, the editors and the reviewers. Any product that may be evaluated in this article, or claim that may be made by its manufacturer, is not guaranteed or endorsed by the publisher.
