# Supplementary material for: Biofilms in Water Hoses of a Meat Processing Environment Harbor Complex Microbial Communities
Source: Front Microbiol. 2022 Feb 14;13:832213. doi: 10.3389/fmicb.2022.832213 (PMC8882869; doi:10.3389/fmicb.2022.832213)
Supplement: Supplementary file 1 [file Data_Sheet_1.PDF]

Supplementary Material

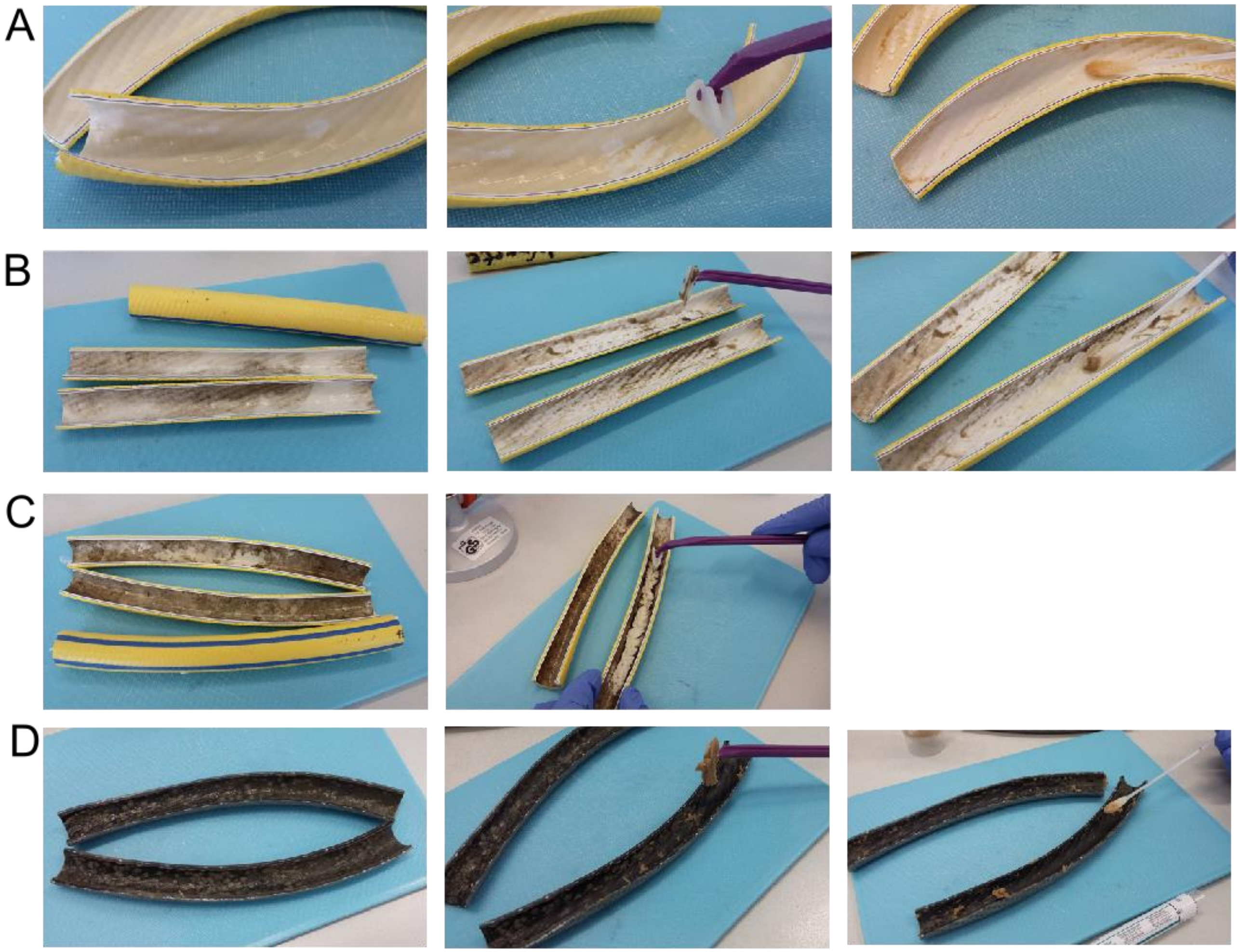

Supplementary Figure 1. Representative images of water hoses

Hoses were cut and the inner biofilm was scraped and swabbed using cell scrapers and swabs. The picture in the first column represents the unprocessed hose, in the second column during scraping and in the third column during swabbing. A=H1, B=H2, C=H6, D=H7.

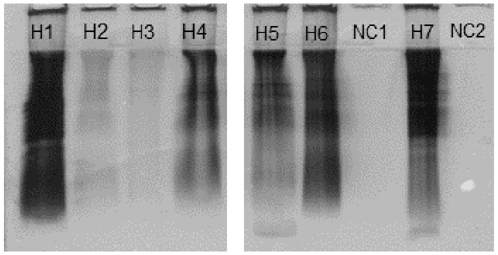

Supplementary Figure 2. Detection of proteins in the biofilm matrix of water hoses using SDS-PAGE and silver-staining.

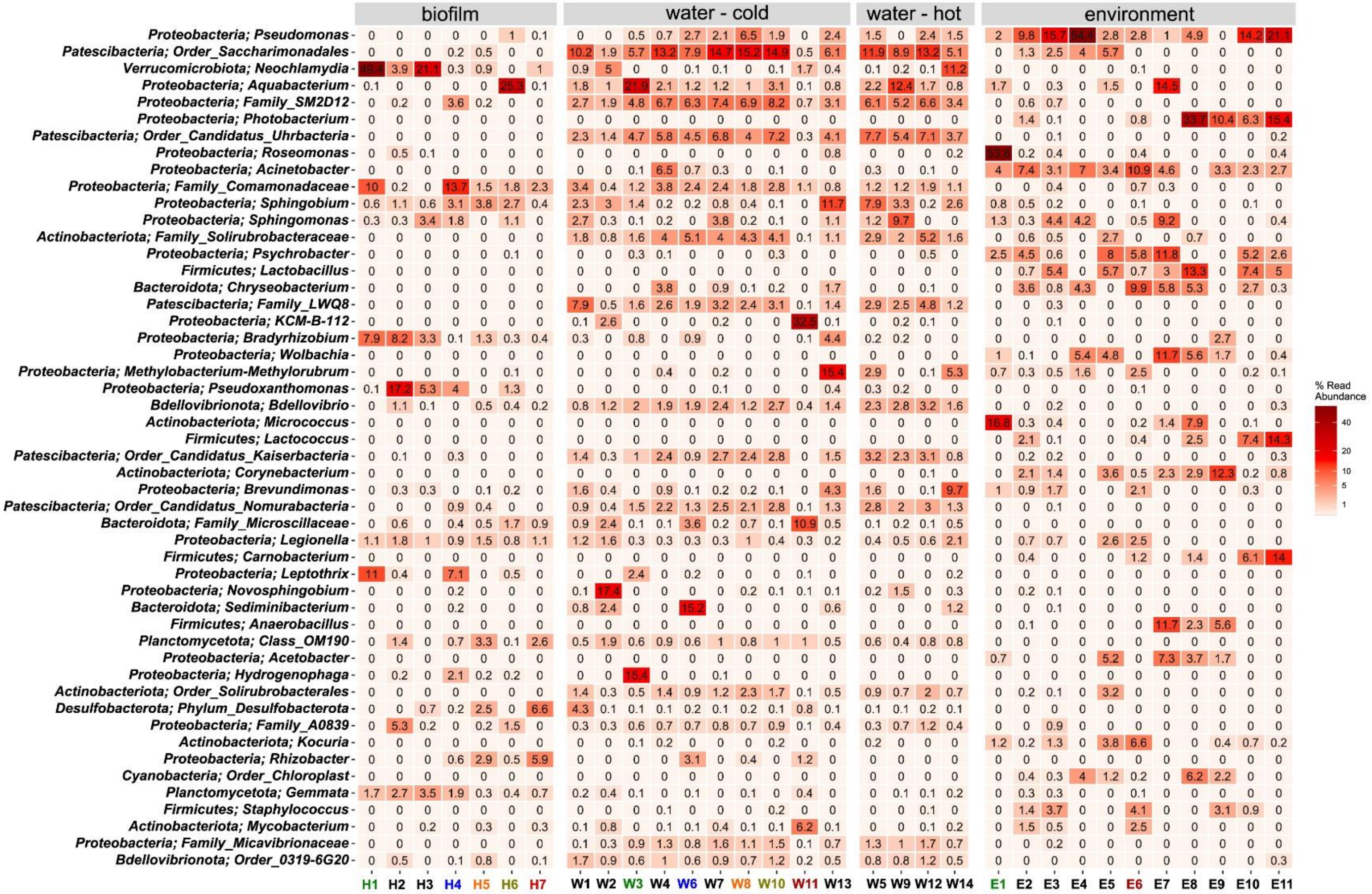

Supplementary Figure 3: Heatmap of the top 50 most abundant genera (and their belonging phylum) split by sample type. The average ASV counts from replicate 16S rRNA gene libraries for each category are shown. Numbers represent the read abundance in %. Same color indicate connected samples e.g. W3-H1-E1

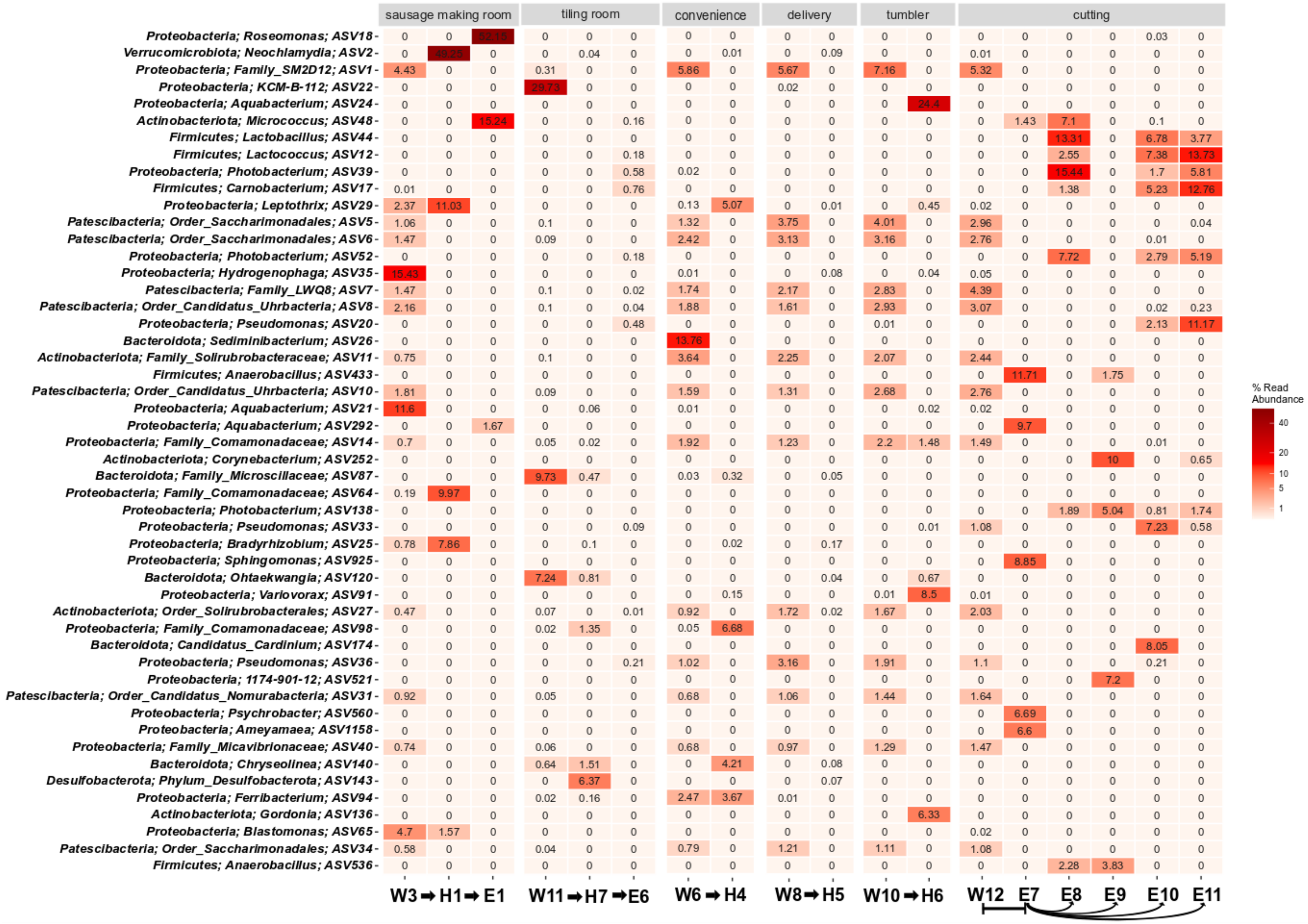

Supplementary Figure 4. Heatmap of the 50 most abundant ASVs within different rooms and individual samples. Arrows indicate water transfer. “E7” is physically attached to W12 influx.

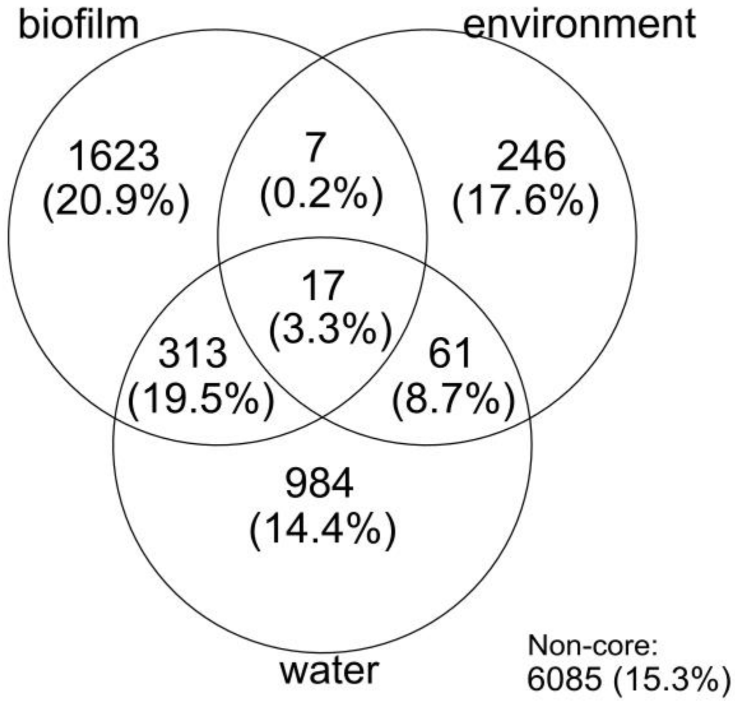

Supplementary Figure 5. Venn diagram showing the numbers (and respective percentage) of shared ASVs between the different sample groups (biofilm, environment, water), as determined by 16S rRNA gene analysis.

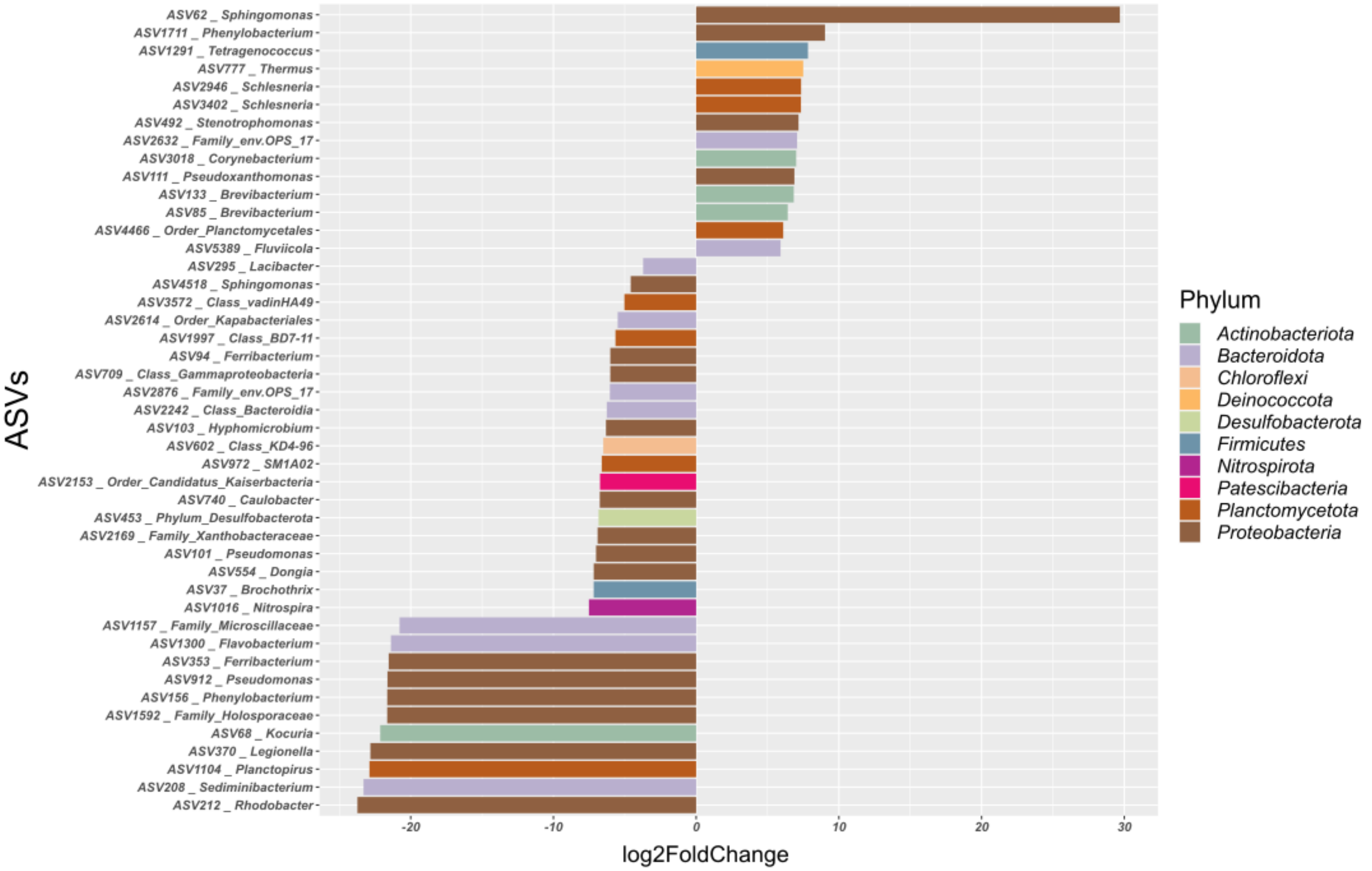

Supplementary Figure 6. Differences between hot and cold water samples.

Barplots of ASVs that were significantly differentially abundant ($p < 0.05$) between hot and cold water samples. Positive values indicate higher abundance in hot water samples and negative values depict higher abundance in cold water samples. Significant ASVs are plotted individually and colored according to their phyla classification.

Supplementary Table 1. Additional information of sampled water hoses and water.

Visible appearance of the biofilms after sampling from the hose and the filters used for collection of the incoming water microbiome.
- no connected sample available.

sample	appearance of sampled biofilm	associated water sample	water (without associated hose)	appearance of filter
H1	turbid / brown particles / brown pellet	W3	-	light yellow
H2	turbid / slightly viscous / brown particles / brown pellet	-	-	-
H3	turbid / slightly viscous / brown particles / brown pellet	-	-	-
H4	turbid / slightly viscous / brown particles / brown pellet	W6	-	light yellow
H5	turbid / yellowish particles	W8	-	yellow-brownish, visible fibers
H6	turbid / brown particles	W10	-	yellow-brownish
H7	turbid / brownish-yellow particles	W11	-	brownish, visible particles
			W1	yellow-brownish, visible fibers
			W2	light yellow, visible fibers
			W4	yellow-brownish, visible fibers
			W5	yellow-brownish, visible fibers
			W7	yellow-brownish
			W9	yellow-brownish
			W12	white
			W13	yellow-brownish, visible particles
			W14	yellow-brownish, visible particles

Supplementary Table 2. Detailed information on environmental samples.

#	FCS/NFCS	room	site	associated with	surface size [cm^2]	used sampling device	BCE/cm^2
E1	FCS	sausage making room	filler	in direct contact with water from H1 derived from W3	50	COPAN swabs	0.47 ± 0.66
E2	FCS	sausage making room	table left	-	100	polyurethane sponges	10.61 ± 0.98
E3	FCS	sausage making room	table right	-	100	polyurethane sponge	19.72 ± 1.47
E4	FCS	convenience room	steaker	-	50	COPAN swabs + scraper	0.22 ± 0.32
E5	FCS	delivery room	packaging table	-	100	COPAN swabs + scraper	0.41 ± 0.59
E6	FCS	tiling room	plating machine	in direct contact with water from H7 derived from W11	200	COPAN swabs + scraper	25.41 ± 5.06
E7	NFCS	cutting room	nozzle of satellite	directly attached to the satellite used for cleaning	2	COPAN swabs	0.00 ± 0.00
E8	NFCS	cutting room	wall	washed by W12 after passing E7	100	COPAN swabs + scraper	1.98 ± 2.81
E9	NFCS	cutting room	wall up right	washed by W12 after passing E7	100	COPAN swabs + scraper	0.53 ± 0.23
E10	FCS	cutting room	table	washed by W12 after passing E7	50	COPAN swabs	123.89 ± 4.71
E11	NFCS	cutting room	table bottom	washed by W12 after passing E7	50	COPAN swabs	14.09 ± 0.01

Supplementary Table 3. Results of the negative controls (NTC).

For water NTC water was retrieved from the MilliQ-aperature and processed as the water samples. For NTC of the hoses all solutions and materials were used as for sampling of the hoses. BCE-values of the respective negative control were subtracted from results of the respective sample type. “-“ analysis not performed, “n.d.” not detectable, “<LOQ” below limit of quantification. Hoses were extracted in two separate runs, for each run one negative control was included.

sample	BCE/cm² or BCE/L	SD BCE	type	comment	carbohydrates [ng/cm²]	eDNA [ng/cm²]	proteins
NTC 1	2.12E+02	8.70E+00	water	cold/hot	-	-	-
NTC 2	0.00E+00	0.00E+00	hose	extraction run 1	< LOQ	< LOQ	n.d.
NTC 3	1.77E+01	2.50E+01	hose	extraction run 2	< LOQ	< LOQ	n.d.
NTC 4	2.44+02	4.85E+01	environment	polyurethane sponge	-	-	-
NTC 5	1.9E+00	4.16E-01	environment	flocked swab	-	-	-

Supplementary Table 4. List of contaminants.

freq	prev	p.prev	p	Kingdom	Phylum	Class	Order	Genus
0.00090	2	0.28889	0.28889	Bacteria	Acidobacteriota	Vicinamibacteria	Subgroup_17	NA
0.00006	2	0.28889	0.28889	Bacteria	Proteobacteria	Alphaproteobacteria	Acetobacterales	Acetobacter
0.00119	15	0.00833	0.00833	Bacteria	Proteobacteria	Alphaproteobacteria	Acetobacterales	Acetobacter
0.00001	2	0.28889	0.28889	Bacteria	Proteobacteria	Alphaproteobacteria	NRL2	NA
0.00001	2	0.28889	0.28889	Bacteria	Proteobacteria	Alphaproteobacteria	NA	NA
0.00012	2	0.28889	0.28889	Bacteria	Proteobacteria	Alphaproteobacteria	Rickettsiales	NA
0.00028	4	0.19605	0.19605	Bacteria	Proteobacteria	Alphaproteobacteria	Rhodobacterales	Amaricoccus
0.00051	7	0.49316	0.49316	Bacteria	Proteobacteria	Gammaproteobacteria	Pseudomonadales	Psychrobacter
0.00001	3	0.42326	0.42326	Bacteria	Proteobacteria	Alphaproteobacteria	Puniceispirillales	NA
0.00011	2	0.28889	0.28889	Bacteria	Proteobacteria	Gammaproteobacteria	Gammaproteobacteria_Incertae_Sedis	Acidibacter
0.00005	2	0.03939	0.03939	Bacteria	Proteobacteria	Alphaproteobacteria	Rhodobacterales	Rhodobacter
0.00022	6	0.39515	0.39515	Bacteria	Actinobacteriota	Actinobacteria	Micrococcales	Brevibacterium
0.00055	6	0.02704	0.02704	Bacteria	Firmicutes	Clostridia	Peptostreptococcales-Tissierellales	Finegoldia
0.00010	4	0.19605	0.19605	Bacteria	Actinobacteriota	Actinobacteria	Micrococcales	Brevibacterium
0.00064	2	0.28889	0.28889	Bacteria	Actinobacteriota	Actinobacteria	Micrococcales	Brevibacterium
0.00058	2	0.03939	0.03939	Bacteria	Actinobacteriota	Actinobacteria	Micrococcales	Brevibacterium
0.00001	3	0.42326	0.42326	Bacteria	Acidobacteriota	Acidobacteriae	Bryobacterales	Bryobacter
0.00021	2	0.28889	0.28889	Bacteria	Acidobacteriota	Acidobacteriae	Bryobacterales	Bryobacter
0.00031	6	0.02704	0.02704	Bacteria	Proteobacteria	Alphaproteobacteria	Rickettsiales	NA
0.00004	2	0.28889	0.28889	Bacteria	Proteobacteria	Alphaproteobacteria	Rickettsiales	NA
0.00003	3	0.42326	0.42326	Bacteria	Acidobacteriota	Blastocatellia	Pyrinomonadales	RB41
0.00213	2	0.28889	0.28889	Bacteria	Acidobacteriota	Blastocatellia	Blastocatellales	NA
0.00004	3	0.42326	0.42326	Bacteria	Acidobacteriota	Blastocatellia	Blastocatellales	JGI_0001001-H03
0.00002	2	0.28889	0.28889	Bacteria	Acidobacteriota	Blastocatellia	Blastocatellales	Blastocatella
0.00001	2	0.28889	0.28889	Bacteria	Acidobacteriota	Blastocatellia	Blastocatellales	Blastocatella
0.00061	3	0.42326	0.42326	Bacteria	Acidobacteriota	Blastocatellia	Blastocatellales	Aridibacter
0.00053	2	0.28889	0.28889	Bacteria	Verrucomicrobiota	Chlamydiae	Chlamydiales	Neochlamydia
0.00043	2	0.28889	0.28889	Bacteria	Verrucomicrobiota	Chlamydiae	Chlamydiales	NA
0.00002	2	0.28889	0.28889	Bacteria	Actinobacteriota	Acidimicrobiia	Microtrichales	NA
0.00001	2	0.28889	0.28889	Bacteria	Actinobacteriota	Acidimicrobiia	IMCC26256	NA
0.00027	2	0.28889	0.28889	Bacteria	Patescibacteria	Microgenomatia	Candidatus_Pacebacteria	NA
0.00003	2	0.28889	0.28889	Bacteria	Dependentiae	Babeliae	Babeliales	NA
0.00001	2	0.28889	0.28889	Bacteria	Dependentiae	Babeliae	Babeliales	NA
0.00014	3	0.42326	0.42326	Bacteria	WPS-2	NA	NA	NA
0.00030	3	0.42326	0.42326	Bacteria	Actinobacteriota	Actinobacteria	Frankiales	Blastococcus
0.00001	3	0.42326	0.42326	Bacteria	Desulfobacterota	NA	NA	NA

0.00002	3	0.42326	0.42326	Bacteria	Gemmatimonadota	Gemmatimonadetes	Gemmatimonadales	NA
0.00002	3	0.42326	0.42326	Bacteria	Bdellovibrionota	Bdellovibrionia	Bdellovibrionales	OM27_clade
0.00045	5	0.29422	0.29422	Bacteria	Firmicutes	Bacilli	Staphylococcales	Staphylococcus
0.00066	10	0.20501	0.20501	Bacteria	Firmicutes	Bacilli	Staphylococcales	Staphylococcus
0.00550	11	0.09824	0.09824	Bacteria	Firmicutes	Bacilli	Staphylococcales	Staphylococcus
0.00257	8	0.09346	0.09346	Bacteria	Firmicutes	Bacilli	Staphylococcales	Staphylococcus
0.00031	2	0.28889	0.28889	Bacteria	Firmicutes	Bacilli	Staphylococcales	Staphylococcus
0.00046	2	0.03939	0.03939	Bacteria	Firmicutes	Bacilli	Staphylococcales	Staphylococcus
0.00261	9	0.37155	0.37155	Bacteria	Firmicutes	Bacilli	Staphylococcales	Staphylococcus
0.00006	2	0.28889	0.28889	Bacteria	Firmicutes	Bacilli	Staphylococcales	Staphylococcus
0.00002	2	0.28889	0.28889	Bacteria	Firmicutes	Bacilli	Staphylococcales	Staphylococcus
0.00015	2	0.28889	0.28889	Bacteria	Firmicutes	Bacilli	Staphylococcales	Staphylococcus
0.00024	2	0.28889	0.28889	Bacteria	Firmicutes	Bacilli	Staphylococcales	Staphylococcus
0.00012	3	0.42326	0.42326	Bacteria	Firmicutes	Bacilli	Staphylococcales	Staphylococcus
0.00021	5	0.29422	0.29422	Bacteria	Cyanobacteria	Cyanobacteriia	Chloroplast	NA
0.00025	3	0.42326	0.42326	Bacteria	Cyanobacteria	Cyanobacteriia	Chloroplast	NA
0.00028	3	0.10810	0.10810	Bacteria	Cyanobacteria	Cyanobacteriia	Chloroplast	NA
0.00011	5	0.29422	0.29422	Bacteria	Cyanobacteria	Cyanobacteriia	Chloroplast	NA
0.00166	15	0.00111	0.00111	Bacteria	Cyanobacteria	Cyanobacteriia	Chloroplast	NA
0.00133	7	0.20678	0.20678	Bacteria	Cyanobacteria	Cyanobacteriia	Chloroplast	NA
0.00001	2	0.28889	0.28889	Bacteria	Actinobacteriota	Actinobacteria	Propionibacteriales	Aeromicrobium
0.00009	3	0.42326	0.42326	Bacteria	Actinobacteriota	Actinobacteria	Propionibacteriales	Marmoricola
0.00004	2	0.28889	0.28889	Bacteria	Proteobacteria	Gammaproteobacteria	Burkholderiales	Massilia
0.00006	2	0.28889	0.28889	Bacteria	Proteobacteria	Gammaproteobacteria	Burkholderiales	Massilia
0.00004	3	0.42326	0.42326	Bacteria	Proteobacteria	Gammaproteobacteria	Burkholderiales	Massilia
0.00242	6	0.02704	0.02704	Bacteria	Proteobacteria	Alphaproteobacteria	Sphingomonadales	Sphingomonas
0.00016	3	0.42326	0.42326	Bacteria	Proteobacteria	Alphaproteobacteria	Sphingomonadales	Blastomonas
0.00009	2	0.28889	0.28889	Bacteria	Proteobacteria	Alphaproteobacteria	Sphingomonadales	Novosphingobium
0.00008	2	0.28889	0.28889	Bacteria	Proteobacteria	Alphaproteobacteria	Sphingomonadales	Sphingomonas
0.00003	3	0.42326	0.42326	Bacteria	Proteobacteria	Alphaproteobacteria	Sphingomonadales	Sphingomonas
0.00007	3	0.42326	0.42326	Bacteria	Proteobacteria	Alphaproteobacteria	Sphingomonadales	NA
0.00017	2	0.28889	0.28889	Bacteria	Proteobacteria	Gammaproteobacteria	Burkholderiales	Comamonas
0.00002	2	0.28889	0.28889	Bacteria	Proteobacteria	Gammaproteobacteria	Burkholderiales	Comamonas
0.02225	26	0.00045	0.00045	Bacteria	Proteobacteria	Gammaproteobacteria	Burkholderiales	Delftia
0.00005	2	0.28889	0.28889	Bacteria	Proteobacteria	Gammaproteobacteria	Burkholderiales	NA
0.00158	10	0.01255	0.01255	Bacteria	Proteobacteria	Gammaproteobacteria	Burkholderiales	Burkholderia-Caballeronia-Paraburkholderia
0.00191	11	0.00370	0.00370	Bacteria	Proteobacteria	Gammaproteobacteria	Burkholderiales	Ralstonia
0.00014	3	0.42326	0.42326	Bacteria	Proteobacteria	Gammaproteobacteria	Salinisphaerales	NA

0.00001	3	0.42326	0.42326	Bacteria	Proteobacteria	Gammaproteobacteria	Burkholderiales	Gallionella
0.00005	2	0.28889	0.28889	Bacteria	Bacteroidota	Bacteroidia	Flavobacteriales	Chryseobacterium
0.00009	2	0.28889	0.28889	Bacteria	Proteobacteria	Alphaproteobacteria	Rickettsiales	Wolbachia
0.00014	2	0.28889	0.28889	Bacteria	Proteobacteria	Alphaproteobacteria	Rickettsiales	Wolbachia
0.03992	26	0.00045	0.00045	Bacteria	Proteobacteria	Alphaproteobacteria	Rickettsiales	Wolbachia
0.00116	4	0.03551	0.03551	Bacteria	Proteobacteria	Alphaproteobacteria	Rhizobiales	Chelativorans
0.00009	2	0.03939	0.03939	Bacteria	Proteobacteria	Alphaproteobacteria	Rhizobiales	Psychroglaciecola
0.00007	2	0.28889	0.28889	Bacteria	Proteobacteria	Alphaproteobacteria	Rickettsiales	NA
0.00002	3	0.42326	0.42326	Bacteria	Proteobacteria	Alphaproteobacteria	Ferrovibrionales	NA
0.00005	2	0.28889	0.28889	Bacteria	Proteobacteria	Gammaproteobacteria	Burkholderiales	Acidovorax
0.00002	3	0.42326	0.42326	Bacteria	Proteobacteria	Gammaproteobacteria	Burkholderiales	Acidovorax
0.00117	16	0.18512	0.18512	Bacteria	Proteobacteria	Gammaproteobacteria	Burkholderiales	Acidovorax
0.00005	3	0.42326	0.42326	Bacteria	Proteobacteria	Gammaproteobacteria	Burkholderiales	Acidovorax
0.00000	3	0.42326	0.42326	Bacteria	Proteobacteria	Gammaproteobacteria	Burkholderiales	Acidovorax
0.00027	2	0.28889	0.28889	Bacteria	Proteobacteria	Gammaproteobacteria	Burkholderiales	Diaphorobacter
0.00025	2	0.28889	0.28889	Bacteria	Proteobacteria	Gammaproteobacteria	Burkholderiales	Acidovorax
0.00014	2	0.28889	0.28889	Bacteria	Proteobacteria	Gammaproteobacteria	Burkholderiales	Curvibacter
0.00015	5	0.29422	0.29422	Bacteria	Proteobacteria	Gammaproteobacteria	Burkholderiales	NA
0.00039	2	0.28889	0.28889	Bacteria	Proteobacteria	Gammaproteobacteria	Pseudomonadales	Acinetobacter
0.00895	23	0.00006	0.00006	Bacteria	Proteobacteria	Gammaproteobacteria	Pseudomonadales	Pseudomonas
0.08978	28	0.00026	0.00026	Bacteria	Proteobacteria	Gammaproteobacteria	Pseudomonadales	Pseudomonas
0.01676	26	0.00045	0.00045	Bacteria	Proteobacteria	Gammaproteobacteria	Pseudomonadales	Pseudomonas
0.00017	4	0.19605	0.19605	Bacteria	Proteobacteria	Gammaproteobacteria	Pseudomonadales	Pseudomonas
0.00051	11	0.09824	0.09824	Bacteria	Proteobacteria	Gammaproteobacteria	Pseudomonadales	Pseudomonas
0.00016	2	0.28889	0.28889	Bacteria	Proteobacteria	Gammaproteobacteria	Pseudomonadales	Pseudomonas
0.00272	20	0.00089	0.00089	Bacteria	Proteobacteria	Gammaproteobacteria	Pseudomonadales	Pseudomonas
0.00059	10	0.20501	0.20501	Bacteria	Proteobacteria	Gammaproteobacteria	Pseudomonadales	Pseudomonas
0.01298	31	0.00141	0.00141	Bacteria	Proteobacteria	Gammaproteobacteria	Pseudomonadales	Pseudomonas
0.00031	6	0.39515	0.39515	Bacteria	Proteobacteria	Gammaproteobacteria	Pseudomonadales	Pseudomonas
0.00807	31	0.08266	0.08266	Bacteria	Proteobacteria	Gammaproteobacteria	Pseudomonadales	Pseudomonas
0.00018	2	0.28889	0.28889	Bacteria	Proteobacteria	Alphaproteobacteria	Caulobacterales	Glycocaulis
0.00149	7	0.49316	0.49316	Bacteria	Firmicutes	Clostridia	Peptostreptococcales-Tissierellales	Romboutsia
0.00004	3	0.42326	0.42326	Bacteria	Firmicutes	Clostridia	Peptostreptococcales-Tissierellales	Romboutsia
0.00018	3	0.42326	0.42326	Bacteria	Actinobacteriota	Actinobacteria	Micrococcales	Curtobacterium
0.00002	2	0.28889	0.28889	Bacteria	Actinobacteriota	Actinobacteria	Micrococcales	Frigoribacterium
0.00014	3	0.42326	0.42326	Bacteria	Actinobacteriota	Actinobacteria	Micrococcales	Dermacoccus
0.00008	3	0.10810	0.10810	Bacteria	Actinobacteriota	Actinobacteria	Micrococcales	Kocuria
0.00002	2	0.28889	0.28889	Bacteria	Actinobacteriota	Actinobacteria	Kineosporiales	Kineococcus
0.00011	5	0.29422	0.29422	Bacteria	Actinobacteriota	Actinobacteria	Micrococcales	Janibacter
0.00017	3	0.42326	0.42326	Bacteria	Actinobacteriota	Actinobacteria	Micrococcales	Micrococcus

0.00005	2	0.28889	0.28889	Bacteria	Actinobacteriota	Actinobacteria	Micrococcales	Brachybacterium
0.00009	3	0.42326	0.42326	Bacteria	Actinobacteriota	Actinobacteria	Micrococcales	Enteractinococcus
0.00005	3	0.42326	0.42326	Bacteria	Actinobacteriota	Actinobacteria	Corynebacteriales	Corynebacterium
0.00003	2	0.28889	0.28889	Bacteria	Actinobacteriota	Actinobacteria	Corynebacteriales	Corynebacterium
0.00010	2	0.28889	0.28889	Bacteria	Actinobacteriota	Actinobacteria	Corynebacteriales	Corynebacterium
0.00006	3	0.42326	0.42326	Bacteria	Actinobacteriota	Actinobacteria	Micrococcales	Brachybacterium
0.00001	2	0.28889	0.28889	Bacteria	Actinobacteriota	Actinobacteria	Corynebacteriales	Mycobacterium
0.00025	3	0.42326	0.42326	Bacteria	Actinobacteriota	Actinobacteria	Micrococcales	Brachybacterium
0.00009	3	0.01008	0.01008	Bacteria	Actinobacteriota	Actinobacteria	Corynebacteriales	Dietzia
0.00031	3	0.42326	0.42326	Bacteria	Firmicutes	Bacilli	Lactobacillales	Leuconostoc
0.00005	2	0.28889	0.28889	Bacteria	Desulfobacterota	Desulfobacteria	Desulfobacterales	Desulfobacter
0.00209	9	0.37155	0.37155	Bacteria	Firmicutes	Bacilli	Lactobacillales	Lactococcus
0.00001	2	0.28889	0.28889	Bacteria	Planctomycetota	Planctomycetes	Pirellulales	Pir4_lineage
0.00096	3	0.42326	0.42326	Bacteria	Acidobacteriota	Holophagae	Holophagales	Geothrix
0.00014	3	0.01008	0.01008	Bacteria	Firmicutes	Bacilli	Bacillales	Anaerobacillus
0.00014	2	0.28889	0.28889	Bacteria	Firmicutes	Bacilli	Staphylococcales	Macrococcus
0.00564	21	0.00173	0.00173	Bacteria	Firmicutes	Bacilli	Lactobacillales	Carnobacterium
0.00111	6	0.39515	0.39515	Bacteria	Firmicutes	Bacilli	Lactobacillales	Carnobacterium
0.00013	2	0.28889	0.28889	Bacteria	Firmicutes	Negativicutes	Veillonellales-Selenomonadales	Zymophilus
0.00015	3	0.42326	0.42326	Bacteria	Firmicutes	Bacilli	Staphylococcales	Jeotgalicoccus
0.00075	5	0.07783	0.07783	Bacteria	Firmicutes	Bacilli	Bacillales	Bacillus
0.00021	3	0.42326	0.42326	Bacteria	Firmicutes	Bacilli	Lactobacillales	Listeria
0.00017	3	0.42326	0.42326	Bacteria	Firmicutes	Bacilli	Lactobacillales	Carnobacterium
0.00227	15	0.00833	0.00833	Bacteria	Firmicutes	Bacilli	Bacillales	Bacillus
0.00001	2	0.28889	0.28889	Bacteria	Patescibacteria	Parcubacteria	NA	NA
0.00015	5	0.29422	0.29422	Bacteria	Firmicutes	Bacilli	Lactobacillales	Lactobacillus
0.00024	3	0.42326	0.42326	Bacteria	Firmicutes	Bacilli	Exiguobacterales	Exiguobacterium
0.00002	2	0.28889	0.28889	Bacteria	Patescibacteria	Saccharimonadia	Saccharimonadales	NA
0.00001	2	0.28889	0.28889	Bacteria	Patescibacteria	Parcubacteria	Candidatus_Kaiserbacteria	NA
0.00009	2	0.28889	0.28889	Bacteria	Spirochaetota	Spirochaetia	Spirochaetales	Sphaerochaeta
0.00004	2	0.28889	0.28889	Bacteria	Synergistota	Synergistia	Synergistales	Aminomonas
0.00002	2	0.28889	0.28889	Bacteria	Actinobacteriota	Thermoleophilia	Solirubrobacterales	NA
0.00003	2	0.28889	0.28889	Bacteria	Bacteroidota	Bacteroidia	Flavobacteriales	Chryseobacterium
0.00014	2	0.28889	0.28889	Bacteria	Bacteroidota	Bacteroidia	Flavobacteriales	Chryseobacterium
0.00031	2	0.28889	0.28889	Bacteria	Firmicutes	Bacilli	Lactobacillales	Atopostipes
0.00001	2	0.28889	0.28889	Bacteria	Planctomycetota	Planctomycetes	Gemmatales	Fimbriiglobus
0.00259	7	0.20678	0.20678	Bacteria	Firmicutes	Bacilli	Lactobacillales	Streptococcus
0.00002	2	0.28889	0.28889	Bacteria	Proteobacteria	Gammaproteobacteria	Legionellales	Legionella
0.00003	3	0.42326	0.42326	Bacteria	Proteobacteria	Gammaproteobacteria	Legionellales	Legionella

0.00190	7	0.49316	0.49316	Bacteria	Proteobacteria	Alphaproteobacteria	NA	NA
0.00007	3	0.42326	0.42326	Bacteria	Proteobacteria	Gammaproteobacteria	Legionellales	Legionella
0.00059	11	0.27423	0.27423	Bacteria	Proteobacteria	Gammaproteobacteria	Burkholderiales	Ralstonia
0.00045	6	0.13589	0.13589	Bacteria	Proteobacteria	Gammaproteobacteria	Burkholderiales	Cupriavidus
0.00040	7	0.20678	0.20678	Bacteria	Proteobacteria	Gammaproteobacteria	Burkholderiales	Cupriavidus
0.00057	7	0.20678	0.20678	Bacteria	Proteobacteria	Alphaproteobacteria	Acetobacterales	Acetobacter
0.00154	17	0.02412	0.02412	Bacteria	Proteobacteria	Alphaproteobacteria	Acetobacterales	Acetobacter
0.00105	14	0.00003	0.00003	Bacteria	Proteobacteria	Alphaproteobacteria	Acetobacterales	Acetobacter
0.00032	9	0.00053	0.00053	Bacteria	Proteobacteria	Alphaproteobacteria	Acetobacterales	Acetobacter
0.00010	3	0.42326	0.42326	Bacteria	Proteobacteria	Alphaproteobacteria	Acetobacterales	Acetobacter
0.00020	2	0.28889	0.28889	Bacteria	Proteobacteria	Alphaproteobacteria	Rhizobiales	Bradyrhizobium
0.00161	4	0.03551	0.03551	Bacteria	Proteobacteria	Alphaproteobacteria	Rhizobiales	Pelagibacterium
0.00005	2	0.28889	0.28889	Bacteria	Proteobacteria	Alphaproteobacteria	Reyranellales	Reyranella
0.00028	2	0.28889	0.28889	Bacteria	Proteobacteria	Alphaproteobacteria	Reyranellales	Reyranella
0.00004	3	0.42326	0.42326	Bacteria	Proteobacteria	Alphaproteobacteria	Reyranellales	Reyranella
0.00129	3	0.42326	0.42326	Bacteria	Proteobacteria	Gammaproteobacteria	Burkholderiales	Piscinibacter
0.00000	2	0.28889	0.28889	Bacteria	Proteobacteria	Gammaproteobacteria	Burkholderiales	NA
0.00069	10	0.06284	0.06284	Bacteria	Proteobacteria	Gammaproteobacteria	Burkholderiales	Schlegelella
0.00001	3	0.42326	0.42326	Bacteria	Proteobacteria	Gammaproteobacteria	Oceanospirillales	Halomonas
0.00042	3	0.42326	0.42326	Bacteria	Proteobacteria	Gammaproteobacteria	Pseudomonadales	Psychrobacter
0.00027	6	0.39515	0.39515	Bacteria	Proteobacteria	Gammaproteobacteria	Pseudomonadales	Psychrobacter
0.00032	3	0.10810	0.10810	Bacteria	Proteobacteria	Gammaproteobacteria	Pseudomonadales	Psychrobacter
0.00001	3	0.10810	0.10810	Bacteria	Proteobacteria	Gammaproteobacteria	Pseudomonadales	Psychrobacter
0.00005	2	0.03939	0.03939	Bacteria	Proteobacteria	Gammaproteobacteria	Oceanospirillales	Halomonas
0.00055	7	0.49316	0.49316	Bacteria	Proteobacteria	Gammaproteobacteria	Burkholderiales	Methyloversatilis
0.00043	5	0.07783	0.07783	Bacteria	Proteobacteria	Gammaproteobacteria	Burkholderiales	Methyloversatilis
0.00012	4	0.19605	0.19605	Bacteria	Proteobacteria	Alphaproteobacteria	Rhodobacterales	Paracoccus
0.00036	7	0.20678	0.20678	Bacteria	Proteobacteria	Alphaproteobacteria	Rhodobacterales	Paracoccus
0.00014	3	0.42326	0.42326	Bacteria	Proteobacteria	Alphaproteobacteria	Rhodobacterales	Paracoccus
0.00153	7	0.49316	0.49316	Bacteria	Proteobacteria	Alphaproteobacteria	Rhodobacterales	Paracoccus
0.00002	2	0.28889	0.28889	Bacteria	Bacteroidota	Bacteroidia	Cytophagales	Spirosoma
0.00002	2	0.28889	0.28889	Bacteria	Bacteroidota	Bacteroidia	Sphingobacteriales	NA
0.00045	3	0.42326	0.42326	Bacteria	Myxococcota	Polyangia	Polyangiales	Sandaracinus
0.00074	3	0.42326	0.42326	Bacteria	Bacteroidota	Bacteroidia	Flavobacteriales	Cloacibacterium
0.00020	2	0.28889	0.28889	Bacteria	Bacteroidota	Bacteroidia	Flavobacteriales	Flavobacterium
0.00000	2	0.28889	0.28889	Bacteria	Myxococcota	Polyangia	Polyangiales	Sandaracinus
0.00001	2	0.28889	0.28889	Bacteria	Planctomycetota	Phycisphaerae	mle1-8	NA
0.00053	2	0.28889	0.28889	Bacteria	Myxococcota	Polyangia	Haliangiales	Haliangium
0.00002	3	0.42326	0.42326	Bacteria	Planctomycetota	Planctomycetes	Pirellulales	Pirellula
0.00002	2	0.28889	0.28889	Bacteria	Planctomycetota	Planctomycetes	Gemmatales	NA

0.00001	3	0.42326	0.42326	Bacteria	Planctomycetota	Planctomycetes	Gemmatales	Fimbriiglobus
0.00002	3	0.42326	0.42326	Bacteria	Acidobacteriota	Vicinamibacteria	Vicinamibacterales	NA
0.00011	2	0.28889	0.28889	Bacteria	Firmicutes	Bacilli	Alicyclobacillales	Alicyclobacillus
0.00002	3	0.42326	0.42326	Bacteria	Deinococcota	Deinococci	Thermales	Meiothermus
0.00004	3	0.42326	0.42326	Bacteria	Proteobacteria	Alphaproteobacteria	Sphingomonadales	Sphingomonas
0.00024	4	0.19605	0.19605	Bacteria	Proteobacteria	Alphaproteobacteria	Sphingomonadales	Sphingomonas
0.00026	3	0.10810	0.10810	Bacteria	Proteobacteria	Alphaproteobacteria	Azospirillales	Skermanella
0.00134	16	0.00229	0.00229	Bacteria	Proteobacteria	Gammaproteobacteria	Burkholderiales	Curvibacter
0.00014	2	0.03939	0.03939	Bacteria	Proteobacteria	Alphaproteobacteria	Acetobacterales	Roseomonas
0.00096	13	0.19705	0.19705	Bacteria	Proteobacteria	Gammaproteobacteria	Xanthomonadales	Stenotrophomonas
0.00005	2	0.28889	0.28889	Bacteria	Proteobacteria	Alphaproteobacteria	Rhizobiales	Nordella
0.00039	3	0.42326	0.42326	Bacteria	Proteobacteria	Alphaproteobacteria	Rhizobiales	Hyphomicrobium
0.00023	2	0.03939	0.03939	Bacteria	Proteobacteria	Alphaproteobacteria	Rhizobiales	Aliihoeflea
0.00001	3	0.42326	0.42326	Bacteria	Proteobacteria	Alphaproteobacteria	Rhizobiales	Aureimonas
0.00003	2	0.28889	0.28889	Bacteria	Proteobacteria	Alphaproteobacteria	Rhizobiales	NA
0.00004	2	0.28889	0.28889	Bacteria	Proteobacteria	Alphaproteobacteria	Rhizobiales	Hyphomicrobium
0.00002	2	0.28889	0.28889	Bacteria	Proteobacteria	Alphaproteobacteria	Rhizobiales	Bosea
0.00002	3	0.42326	0.42326	Bacteria	Proteobacteria	Alphaproteobacteria	Rhizobiales	Methylobacterium-Methylorubrum
0.00004	3	0.42326	0.42326	Bacteria	Proteobacteria	Alphaproteobacteria	Rhizobiales	Methylobacterium-Methylorubrum
0.00011	2	0.28889	0.28889	Bacteria	Bacteroidota	Bacteroidia	Flavobacteriales	Flavobacterium
0.00120	9	0.37155	0.37155	Bacteria	Proteobacteria	Gammaproteobacteria	Burkholderiales	Acidovorax
0.00013	3	0.42326	0.42326	Bacteria	Proteobacteria	Gammaproteobacteria	Burkholderiales	Acidovorax
0.00062	8	0.01902	0.01902	Bacteria	Proteobacteria	Gammaproteobacteria	Burkholderiales	Variovorax
0.00015	3	0.42326	0.42326	Bacteria	Bacteroidota	Bacteroidia	Flavobacteriales	Flavobacterium
0.00527	20	0.00089	0.00089	Bacteria	Proteobacteria	Gammaproteobacteria	Burkholderiales	Pelomonas
0.00034	6	0.13589	0.13589	Bacteria	Proteobacteria	Gammaproteobacteria	Burkholderiales	Pelomonas
0.00034	4	0.19605	0.19605	Bacteria	Proteobacteria	Gammaproteobacteria	Burkholderiales	Leptothrix
0.00001	2	0.28889	0.28889	Bacteria	Proteobacteria	Gammaproteobacteria	Burkholderiales	Methylibium
0.00002	2	0.28889	0.28889	Bacteria	Proteobacteria	Gammaproteobacteria	Burkholderiales	NA
0.00065	2	0.28889	0.28889	Bacteria	Proteobacteria	Gammaproteobacteria	Burkholderiales	NA
0.00054	4	0.19605	0.19605	Bacteria	Proteobacteria	Gammaproteobacteria	Xanthomonadales	Lysobacter
0.00004	2	0.28889	0.28889	Bacteria	Proteobacteria	Gammaproteobacteria	Alteromonadales	Rheinheimera
0.00002	3	0.42326	0.42326	Bacteria	Proteobacteria	Gammaproteobacteria	Xanthomonadales	Lysobacter
0.00005	2	0.28889	0.28889	Bacteria	Proteobacteria	Gammaproteobacteria	Pseudomonadales	Acinetobacter
0.00010	3	0.42326	0.42326	Bacteria	Proteobacteria	Gammaproteobacteria	Pseudomonadales	Acinetobacter
0.00074	7	0.49316	0.49316	Bacteria	Proteobacteria	Gammaproteobacteria	Pseudomonadales	Acinetobacter
0.00100	9	0.37155	0.37155	Bacteria	Proteobacteria	Gammaproteobacteria	Pseudomonadales	Acinetobacter

0.00007	2	0.28889	0.28889	Bacteria	Proteobacteria	Alphaproteobacteria	Rhizobiales	Allorhizobium-Neorhizobium-Pararhizobium-Rhizobium
0.00016	3	0.42326	0.42326	Bacteria	Proteobacteria	Alphaproteobacteria	Rhizobiales	Xanthobacter
0.00005	2	0.28889	0.28889	Bacteria	Proteobacteria	Gammaproteobacteria	NA	NA
0.00003	2	0.28889	0.28889	Bacteria	Proteobacteria	Alphaproteobacteria	Rhodobacterales	Rhodobacter
0.00009	2	0.28889	0.28889	Bacteria	Proteobacteria	Alphaproteobacteria	Rhodobacterales	Rubellimicrobium
0.00010	3	0.42326	0.42326	Bacteria	Actinobacteriota	Actinobacteria	Micrococcales	Galbitalea
0.00040	2	0.03939	0.03939	Bacteria	Actinobacteriota	Actinobacteria	Micrococcales	Leucobacter
0.00001	2	0.28889	0.28889	Bacteria	Actinobacteriota	Actinobacteria	Micrococcales	Leucobacter
0.00032	2	0.03939	0.03939	Bacteria	Actinobacteriota	Actinobacteria	Micrococcales	Leucobacter
0.00006	3	0.01008	0.01008	Bacteria	Actinobacteriota	Actinobacteria	Propionibacteriales	Cutibacterium
0.00003	2	0.28889	0.28889	Bacteria	Actinobacteriota	Actinobacteria	Propionibacteriales	Friedmanniella
0.00002	2	0.28889	0.28889	Bacteria	Proteobacteria	Gammaproteobacteria	Enterobacterales	Providencia
0.00010	2	0.28889	0.28889	Bacteria	Proteobacteria	Gammaproteobacteria	Enterobacterales	Proteus
0.00024	2	0.28889	0.28889	Bacteria	Proteobacteria	Gammaproteobacteria	Enterobacterales	Proteus
0.00031	3	0.01008	0.01008	Bacteria	Proteobacteria	Gammaproteobacteria	Enterobacterales	Proteus
0.00050	6	0.02704	0.02704	Bacteria	Proteobacteria	Gammaproteobacteria	Enterobacterales	NA
0.00017	2	0.28889	0.28889	Bacteria	Proteobacteria	Gammaproteobacteria	Enterobacterales	Citrobacter
0.00011	3	0.42326	0.42326	Bacteria	Proteobacteria	Gammaproteobacteria	Enterobacterales	Pantoea
0.00011	3	0.42326	0.42326	Bacteria	Proteobacteria	Gammaproteobacteria	Enterobacterales	Enterobacter
0.00139	10	0.20501	0.20501	Bacteria	Proteobacteria	Gammaproteobacteria	Enterobacterales	Escherichia/Shigella
0.00023	3	0.10810	0.10810	Bacteria	Proteobacteria	Gammaproteobacteria	Enterobacterales	Morganella
0.01075	22	0.00036	0.00036	Bacteria	Proteobacteria	Gammaproteobacteria	Enterobacterales	Serratia
0.00501	23	0.00070	0.00070	Bacteria	Proteobacteria	Gammaproteobacteria	Enterobacterales	Serratia
0.02151	26	0.00045	0.00045	Bacteria	Proteobacteria	Gammaproteobacteria	Enterobacterales	Serratia
0.00003	2	0.28889	0.28889	Bacteria	Actinobacteriota	Actinobacteria	Micrococcales	Cellulomonas
0.00095	5	0.07783	0.07783	Bacteria	Actinobacteriota	Actinobacteria	Corynebacteriales	Mycobacterium
0.00012	2	0.28889	0.28889	Bacteria	Actinobacteriota	Actinobacteria	Corynebacteriales	Mycobacterium
0.00011	3	0.42326	0.42326	Bacteria	Actinobacteriota	Actinobacteria	Micrococcales	Rothia
0.00004	2	0.28889	0.28889	Bacteria	Actinobacteriota	Actinobacteria	Corynebacteriales	Gordonia
0.00038	3	0.10810	0.10810	Bacteria	Actinobacteriota	Actinobacteria	Micrococcales	NA
0.00203	13	0.19705	0.19705	Bacteria	Actinobacteriota	Actinobacteria	Micrococcales	Micrococcus
0.00012	2	0.28889	0.28889	Bacteria	Actinobacteriota	Actinobacteria	Micrococcales	Citricoccus
0.00011	4	0.19605	0.19605	Bacteria	Actinobacteriota	Actinobacteria	Corynebacteriales	Mycobacterium
0.00041	5	0.07783	0.07783	Bacteria	Actinobacteriota	Actinobacteria	Corynebacteriales	Mycobacterium
0.00020	4	0.03551	0.03551	Bacteria	Actinobacteriota	Actinobacteria	Micrococcales	Nesterenkonia
0.00033	3	0.42326	0.42326	Bacteria	Actinobacteriota	Actinobacteria	Micrococcales	Nesterenkonia
0.00151	2	0.03939	0.03939	Bacteria	Actinobacteriota	Actinobacteria	Micrococcales	Garicola
0.00017	3	0.42326	0.42326	Bacteria	Actinobacteriota	Actinobacteria	Bifidobacteriales	Bifidobacterium
0.00044	7	0.49316	0.49316	Bacteria	Actinobacteriota	Actinobacteria	Corynebacteriales	Corynebacterium

0.00008	3	0.42326	0.42326	Bacteria	Actinobacteriota	Actinobacteria	Corynebacteriales	Corynebacterium
0.00035	3	0.42326	0.42326	Bacteria	Actinobacteriota	Actinobacteria	Corynebacteriales	Mycobacterium
0.00417	2	0.03939	0.03939	Bacteria	Actinobacteriota	Actinobacteria	Corynebacteriales	Corynebacterium
0.00017	3	0.42326	0.42326	Bacteria	Actinobacteriota	Actinobacteria	Corynebacteriales	Corynebacterium
0.00067	5	0.29422	0.29422	Bacteria	Actinobacteriota	Actinobacteria	Corynebacteriales	Corynebacterium
0.00042	7	0.49316	0.49316	Bacteria	Actinobacteriota	Actinobacteria	Corynebacteriales	Corynebacterium
0.00029	2	0.28889	0.28889	Bacteria	Chloroflexi	Anaerolineae	SBR1031	NA
0.00005	3	0.42326	0.42326	Bacteria	Actinobacteriota	Actinobacteria	Micrococcales	Nesterenkonia
0.00008	2	0.28889	0.28889	Bacteria	Deinococcota	Deinococci	Deinococcales	Deinococcus
0.00039	5	0.07783	0.07783	Bacteria	Firmicutes	Bacilli	Lactobacillales	Enterococcus
0.00007	3	0.10810	0.10810	Bacteria	Firmicutes	Bacilli	Lactobacillales	Lactobacillus
0.00004	2	0.28889	0.28889	Bacteria	Firmicutes	Bacilli	Lactobacillales	Pediococcus
0.00182	2	0.03939	0.03939	Bacteria	Firmicutes	Bacilli	Lactobacillales	Lactobacillus
0.00062	3	0.42326	0.42326	Bacteria	Firmicutes	Bacilli	Lactobacillales	Lactobacillus
0.00003	2	0.28889	0.28889	NA	NA	NA	NA	NA
0.00001	7	0.05439	0.05439	Bacteria	Proteobacteria	Gammaproteobacteria	Pseudomonadales	Pseudomonas
0.00000	4	0.03551	0.03551	Bacteria	Proteobacteria	Alphaproteobacteria	Rickettsiales	Wolbachia
0.00002	2	0.28889	0.28889	Bacteria	Proteobacteria	Gammaproteobacteria	Legionellales	Legionella
0.00019	2	0.28889	0.28889	Bacteria	Proteobacteria	Gammaproteobacteria	Legionellales	Legionella
0.00025	5	0.07783	0.07783	Bacteria	Proteobacteria	Gammaproteobacteria	Pseudomonadales	Acinetobacter
0.00106	5	0.29422	0.29422	Bacteria	Proteobacteria	Gammaproteobacteria	Pseudomonadales	Acinetobacter
0.00003	3	0.42326	0.42326	Bacteria	Proteobacteria	Alphaproteobacteria	Acetobacterales	NA
0.00017	2	0.28889	0.28889	Bacteria	Bacteroidota	Bacteroidia	Flavobacteriales	Empedobacter
0.00010	2	0.28889	0.28889	Bacteria	Proteobacteria	Gammaproteobacteria	Pseudomonadales	Acinetobacter
0.00003	4	0.19605	0.19605	Bacteria	Actinobacteriota	Actinobacteria	Micrococcales	Microbacterium
0.00009	6	0.00274	0.00274	Bacteria	Actinobacteriota	Actinobacteria	Micrococcales	Microbacterium
0.00002	2	0.28889	0.28889	Bacteria	Actinobacteriota	Actinobacteria	Propionibacteriales	Luteococcus
0.00065	22	0.00036	0.00036	Bacteria	Actinobacteriota	Actinobacteria	Propionibacteriales	Cutibacterium
0.00026	6	0.39515	0.39515	Bacteria	Actinobacteriota	Actinobacteria	Corynebacteriales	Lawsonella
0.00103	12	0.00775	0.00775	Bacteria	Actinobacteriota	Actinobacteria	Corynebacteriales	Lawsonella
0.00012	3	0.42326	0.42326	Bacteria	Actinobacteriota	Actinobacteria	Micrococcales	Yaniella
0.00013	5	0.29422	0.29422	Bacteria	Actinobacteriota	Actinobacteria	Corynebacteriales	Dietzia
0.00065	7	0.20678	0.20678	Bacteria	Actinobacteriota	Actinobacteria	Corynebacteriales	Corynebacterium
0.00057	6	0.13589	0.13589	Bacteria	Firmicutes	Bacilli	Lactobacillales	Streptococcus
0.00380	28	0.00026	0.00026	Bacteria	Deinococcota	Deinococci	Thermales	Thermus
0.00000	3	0.42326	0.42326	Bacteria	Cyanobacteria	Cyanobacteriia	Chloroplast	NA
0.00001	7	0.00064	0.00064	Bacteria	Proteobacteria	Gammaproteobacteria	Pseudomonadales	Pseudomonas
0.00000	3	0.42326	0.42326	Bacteria	Firmicutes	Bacilli	Staphylococcales	Staphylococcus
0.00002	4	0.00240	0.00240	Bacteria	Proteobacteria	Alphaproteobacteria	Rickettsiales	Wolbachia

0.00010	2	0.28889	0.28889	Bacteria	Bacteroidota	Bacteroidia	Flavobacteriales	Arenibacter

Supplementary Table 5. Significances of group-wise comparisons of the alpha diversity levels as determined using vegan v2.5-6 (Oksanen et al., 2019) in R.

Alpha diversity index	Group 1	Group 2	p	p.adjusted	p.format	p.signifance	method
observed	biofilm	water - cold	0.003	0.006	0.003	**	Wilcoxon
observed	biofilm	water - hot	0.024	0.036	0.024	*	Wilcoxon
observed	biofilm	environment	0.246	0.300	0.246	ns	Wilcoxon
observed	water - cold	water - hot	0.454	0.450	0.454	ns	Wilcoxon
observed	water - cold	environment	0.000	0.000	0.000	****	Wilcoxon
observed	water - hot	environment	0.001	0.004	0.002	**	Wilcoxon
Shannon	biofilm	water - cold	0.088	0.130	0.088	ns	Wilcoxon
Shannon	biofilm	water - hot	0.073	0.130	0.073	ns	Wilcoxon
Shannon	biofilm	environment	0.479	0.540	0.479	ns	Wilcoxon
Shannon	water - cold	water - hot	0.539	0.540	0.539	ns	Wilcoxon
Shannon	water - cold	environment	0.024	0.130	0.024	*	Wilcoxon
Shannon	water - hot	environment	0.056	0.130	0.056	ns	Wilcoxon
Simspon	biofilm	water - cold	0.364	0.550	0.360	ns	Wilcoxon
Simspon	biofilm	water - hot	0.230	0.460	0.230	ns	Wilcoxon
Simspon	biofilm	environment	0.791	0.790	0.790	ns	Wilcoxon
Simspon	water - cold	water - hot	0.539	0.650	0.540	ns	Wilcoxon
Simspon	water - cold	environment	0.223	0.460	0.220	ns	Wilcoxon
Simspon	water - hot	environment	0.177	0.460	0.180	ns	Wilcoxon

ACE	biofilm	water – cold	0.001	0.004	0.001	**	Wilcoxon
ACE	biofilm	water - hot	0.006	0.009	0.006	**	Wilcoxon
ACE	biofilm	environment	0.315	0.370	0.315	ns	Wilcoxon
ACE	water - cold	water - hot	0.374	0.370	0.374	ns	Wilcoxon
ACE	water - cold	environment	0.000	0.000	0.000	****	Wilcoxon
ACE	water - hot	environment	0.002	0.004	0.002	**	Wilcoxon

Supplementary Table 6. List of the 17 shared ASVs, as indicated in Supplementary Figure 4.

ASV	Kingdom	Phylum	Class	Order	Family	Genus	Species
ASV101	Bacteria	Proteobacteria	Gammaproteobacteria	Pseudomonadales	Pseudomonadaceae	Pseudomonas	Genus_Pseudomonas
ASV116	Bacteria	Dadabacteria	Dadabacteriia	Dadabacteriales	Order_Dadabacteriales	Order_Dadabacteriales	Order_Dadabacteriales
ASV125	Bacteria	Planctomycetota	Planctomycetes	Planctomycetales	Gimesiaceae	Family_Gimesiaceae	Family_Gimesiaceae
ASV14	Bacteria	Proteobacteria	Gammaproteobacteria	Burkholderiales	Comamonadaceae	Family_Comamonadaceae	Family_Comamonadaceae
ASV19	Bacteria	Proteobacteria	Alphaproteobacteria	Sphingomonadales	Sphingomonadaceae	Sphingobium	Xenophagum
ASV23	Bacteria	Proteobacteria	Alphaproteobacteria	Rhizobiales	Beijerinckiaceae	Methylobacterium-Methylorubrum	Genus_Methylobacterium-Methylorubrum
ASV27	Bacteria	Actinobacteriota	Thermoleophilia	Solirubrobacterales	Order_Solirubrobacterales	Order_Solirubrobacterales	Order_Solirubrobacterales
ASV3	Bacteria	Proteobacteria	Gammaproteobacteria	Pseudomonadales	Moraxellaceae	Psychrobacter	Genus_Psychrobacter
ASV304	Bacteria	Proteobacteria	Alphaproteobacteria	Caulobacterales	Caulobacteraceae	Brevundimonas	Genus_Brevundimonas
ASV362	Bacteria	Actinobacteriota	Thermoleophilia	Gaiellales	Gaiellaceae	Gaiella	Genus_Gaiella
ASV393	Bacteria	Proteobacteria	Alphaproteobacteria	Caulobacterales	Caulobacteraceae	Brevundimonas	Genus_Brevundimonas
ASV41	Bacteria	Proteobacteria	Alphaproteobacteria	Caulobacterales	Caulobacteraceae	Brevundimonas	Genus_Brevundimonas
ASV56	Bacteria	Bacteroidota	Bacteroidia	Chitinophagales	Chitinophagaceae	Family_Chitinophagaceae	Family_Chitinophagaceae
ASV600	Bacteria	Proteobacteria	Gammaproteobacteria	Burkholderiales	Oxalobacteraceae	Janthinobacterium	Genus_Janthinobacterium
ASV80	Bacteria	Planctomycetota	Planctomycetes	Pirellulales	Pirellulaceae	Pir4_lineage	Genus_Pir4_lineage
ASV853	Bacteria	Proteobacteria	Gammaproteobacteria	Burkholderiales	Rhodocyclaceae	Azospira	Oryzae
ASV985	Bacteria	Proteobacteria	Gammaproteobacteria	Legionellales	Legionellaceae	Legionella	Genus_Legionella
